# PD-1 receptor deficiency enhances CD30^+^ T_reg_ cell function in melanoma

**DOI:** 10.1038/s41590-025-02172-0

**Published:** 2025-06-02

**Authors:** Jing Xuan Lim, Tegan McTaggart, Seol Kyoung Jung, Katie J. Smith, Gillian Hulme, Stephanie Laba, Yun Qi Ng, Amelia Williams, Rafiqul Hussain, Jonathan Coxhead, Ioana Cosgarea, Catherine Arden, Jérémie Nsengimana, Penny Lovat, Graham Anderson, Hong-Wei Sun, Arian Laurence, Shoba Amarnath

**Affiliations:** 1 Biosciences Institute, Newcastle University, Newcastle University, Newcastle upon Tyne, UK; 2 NIHR, Biomedical Research Centre, Newcastle University, Newcastle upon Tyne, UK; 3 Centre for Cancer Research, Newcastle University, Newcastle upon Tyne, UK; 4 National Institute of Arthritis, Musculoskeletal and Skin, NIH, Bethesda, MD USA; 5 Translational and Clinical Research Institute, Newcastle University, Newcastle upon Tyne, UK; 6 Population Health Sciences Institute, Newcastle University, Newcastle upon Tyne, UK; 7 Department of Immunology and Immunotherapy, University of Birmingham, Birmingham, UK; 8 Translational Gastroenterology Unit, Nuffield School of Medicine, Oxford University, Oxford, UK

**Keywords:** Immunotherapy, Cancer, Regulatory T cells

## Abstract

Regulatory T (T_reg_) cells are vital for immune suppression. The role of the coreceptor programmed cell death 1 receptor (PD-1) in T_reg_ cell function is controversial. Here, we demonstrate that PD-1 deficiency enhances the function of T_reg_ cells through expression of a compensatory network of coinhibitory receptors. CD30 has a central role within this network, driving the T_reg_ cell suppressive function within the tumor microenvironment. Mechanistically, PD-1 deficiency enhances STAT5 signaling in T_reg_ cells, which induces CD30 expression. These data indicate a role for PD-1 as a checkpoint that negatively controls CD30 expression in T_reg_ cells to limit their suppressive function. Understanding the functional changes that PD-1 has on T_reg_ cells might enable combination therapies with better treatment outcomes in cancer.

## Abstract (web summary)

Here the authors show that PD-1 controls coinhibitory receptor expression by T_reg_ cells. The absence of PD-1 expression could induce CD30 expression, thereby enhancing T_reg_ function and tumor escape, suggesting that CD30 might be a therapeutic target in cases of anti-PD-1 resistance.

## Main

Regulatory T (T_reg_) cells are a subset of CD4^+^ T helper cells that restrain the immune system. T_reg_ cells express the forkhead transcription factor FoxP3 (ref. ^1^), along with several coinhibitory receptors, namely cytotoxic T lymphocyte antigen 4 (CTLA4) and programmed cell death 1 receptor (PD-1), to which specific functions have been attributed^2–4^. PD-1 binds to its cognate ligand programmed death ligand 1 (PD-L1) or PD-L2 (ref. ^5^). When bound to PD-L1, PD-1 enhances FoxP3 expression in stimulated murine CD4^+^ T helper cells^3^ and maintains FoxP3 expression in human and murine Tbet^+^ T helper 1 cells^4,6^.

PD-1 and PD-L1 antibodies have been used successfully in the treatment of several cancers, yet many cancer patients do not respond to this treatment, and in some cases PD-1 inhibition may enhance cancer growth. The underlying mechanisms that drive this resistance within the immune system are unclear. Correlative studies in human cancers have shown that immune resistance after PD-1 therapy can be attributed to T_reg_ cells,^7^ and PD-1 deficiency drives T_reg_ cell dysfunction^8^. Current observations^9–11^ are in line with findings by Chen et al.^12^ that PD-1 deficiency has an impact on peripheral T_reg_ cell generation. Hence, PD-1 can negatively affect T_reg_ cell function in autoimmunity and infections, potentially contributing to immunotherapy resistance in cancer and driving hyperprogressive disease^7,10^. Therefore, the current literature reports both negative and positive regulatory functions for PD-1 in T_reg_ cells, with a lack of consensus on the role of PD-1 in driving T_reg_ cell function.

Understanding of PD-1 signaling in T_reg_ cells might provide insight into its function. The downstream signaling of PD-1 is dependent on its ITSM motif^13^, which recruits and activates Src homology phosphatase 1/2 (SHP1/2)^14,15^, leading to dephosphorylation and inactivation of downstream signaling pathways. PD-1 signaling in CD8^+^ T cells and (to some extent) in T_reg_ cells has been shown to inhibit downstream TCR and CD28 signaling^16,17^. This includes inhibition of the PI3K–Akt signaling pathway in naive murine T cells, which drives functional plasticity of these cells and their differentiation into T_reg_ cells^3^. However, the opposite has also been shown to enhance T_reg_ cell function, with unchecked PI3K–Akt signaling enhancing the proliferation of T_reg_ cells, leading to superior function in the absence of PD-1 (ref. ^9^).

We have previously found that PD-1 can dampen STAT1/4/5 phosphorylation^4,18^, and PD-1–STAT5 signaling in T_reg_ cells has been reported to be important in human viral infections^19,20^. The outcome of enhanced STAT5 signaling in T_reg_ cells is unclear; suggesting a crucial role for enhanced STAT5 in driving a regulatory phenotype in T_reg_ cells, independent of FoxP3 expression.

In this study, we use BD Rhapsody mouse and human single-cell RNA sequencing (scRNA-seq) analysis and 1000-plex CosMx spatial analysis to identify an alternative regulatory pathway in PD-1-deficient T_reg_ cells that can enhance their function. In the absence of PD-1, an increase in specific coinhibitory receptor expression is noted that enhances T_reg_ cell function within the tumor microenvironment (TME). Mechanistically, PD-1 deficiency enhances STAT5 signaling, which in turn results in upregulation of CD30 receptor on T_reg_ cells, driving increased T_reg_ cell function. We confirm that CD30 expression in human T_reg_ cells is driven by blockade of the PD-1–PD-L1 pathway. These findings highlight the importance of PD-1 in T_reg_ cell function and suggest combination strategies that could benefit patients who are refractory to PD-1 therapy owing to increased T_reg_ cell numbers.

## Results

### PD-1 intrinsically controls CD30 expression by T_reg_ cells

**Fig. 1:**
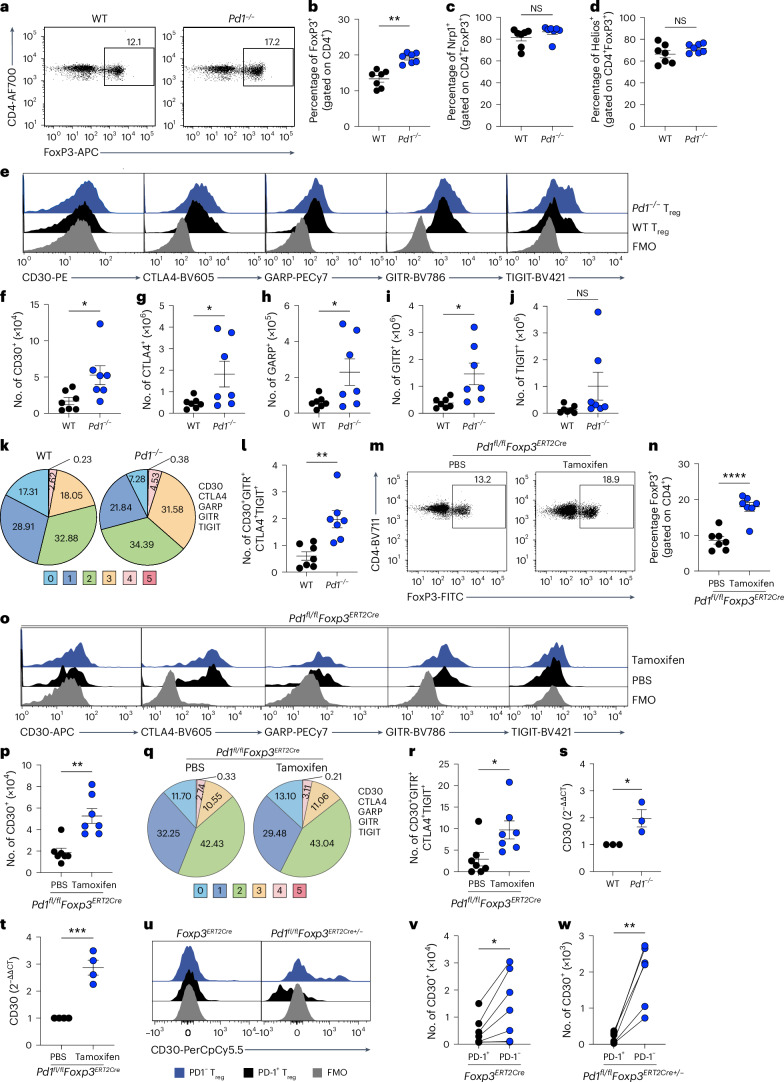
PD-1 deficiency in T_reg_ cells upregulates coinhibitory receptors including CD30. **a**–**j**, *Foxp3*^*RFP*^ (WT) and *Pd1*^*−/−*^*Foxp3*^*RFP*^ (*Pd1*^*−/−*^) spleens were immunophenotyped using flow cytometry (*n* = 7 per group): flow cytometry profiles (**a**) and summaries of FoxP3 expression (**b**) in WT and *Pd1*^*−/−*^ mice; frequencies of neuropilin 1 (Nrp1) (**c**) and Helios (**d**) expression in T_reg_ cells; flow cytometry profiles of CD30, CTLA4, GARP, GITR and TIGIT expression in T_reg_ cells (**e**); and absolute numbers of CD30 (**f**), CTLA4 (**g**), GARP (**h**), GITR (**i**) and TIGIT (**j**) in T_reg_ cells are shown. **k**, Boolean analysis of expression of five different coinhibitory receptors in T_reg_ cells. **l**, Linear analysis of CD30 network of coreceptors in T_reg_ cells. **m**–**r**, Spleens of *Pd1*^*fl/fl*^*Foxp3*^*ERT2Cre*^ mice that had undergone preadministration of 1 mg tamoxifen or PBS for 5 consecutive days via i.p. injection (*n* = 7 per group) were analyzed: representative flow cytometry analysis of FoxP3 expression (**m**); summary data of FoxP3 expression (**n**); a representative flow plot of CD30, CTLA4, GARP, GITR and TIGIT expression in T_reg_ cells (**o**); absolute numbers of CD30 in T_reg_ cells (**p**); Boolean analysis of expression of five different coinhibitory receptors in T_reg_ cells (**q**); and absolute counts of the CD30 network of coreceptors (**r**) are shown. **s**,**t**, RT–qPCR analysis of *Tnfrsf8* mRNA expression in T_reg_ cells from *Pd1*^*−/−*^*Foxp3*^*RFP*^ (*n* = 7) (**s**) and *Pd1*^*fl/fl*^*Foxp3*^*ERT2Cre+/−*^ (*n* = 6) (**t**) female mice treated with tamoxifen. **u**, Representative flow cytometry analysis of CD30 expression in CD4^+^FoxP3^+^CD44^+^PD-1^+^ and CD4^+^FoxP3^+^CD44^+^PD-1^−^ T cells. **v**,**w**, Absolute numbers of CD30 in PD-1^+^ and PD-1^−^ T_reg_ cells from *Foxp3*^*ERT2Cre*^ (**v**) and *Pd1*^*fl/fl*^*Foxp3*^*ERT2Cre+/−*^ (**w**) mice. Data are shown as mean ± s.e.m., with each data point representing an animal from an independent experiment, except in **v** and **w**, in which each pair of data points are from the same animal’s T_reg_ cells with or without PD-1 expression and from independent experiments. Two-tailed unpaired *S*tudent’s *t*-tests were performed for the results shown in **b**–**d**, **f**–**j**, **l**, **n**, **p** and **r**–**t** and two-tailed paired Student’s *t*-tests for those shown in **v** and **w**. *P < 0.05, **P < 0.01, ***P < 0.001, ****P < 0.0001. FMO, fluorescence minus one; NS, not significant.

A role of PD-1 in modifying T_reg_ cell frequency in steady state has been reported^9^, but the expression and function of other coinhibitory receptors within these T_reg_ cells has not been explored. We found a significant increase in FoxP3 frequency in splenocytes from *Pd1*^*−/−*^*Foxp3*^*RFP*^ mice compared with wild-type (WT) animals (note that *Pd1* has been used here in place of the official gene symbol *Pdcd1*) (Fig. 1a,b and Supplementary Fig. 1a). No difference was noted in the proportion of T_reg_ cells that expressed Nrp1, Helios, CD25, Tbet, Gata3 or RORγt^21–23^ (Fig. 1c,d and Supplementary Fig. 1b–f). We investigated the expression of CD30, CTLA4, GARP, GITR and TIGIT coinhibitory receptors on WT and *Pd1*^*−/−*^ T_reg_ cell subsets and found significant increases in absolute numbers of all measured coreceptors except TIGIT in the cells from *Pd1*^*−/−*^ mice compared with WT mice (Fig. 1e–j). Following Odorizzi et al.^24^, we performed Boolean analysis of coinhibitory receptor networks and found a significant increase in the coinhibitory receptor network CD30^+^GITR^+^CTLA4^+^TIGIT^+^ subset in *Pd1*^*−/−*^ T_reg_ cells (Fig. 1k,l and Supplementary Table 1). No change in frequency or coreceptor landscape was noted in CD4^+^FoxP3^*−*^ cells, suggesting specific regulation driven by PD-1 in T_reg_ cells (Supplementary Fig. 1g–i). Similar to Perry et al.^11^, we observed an increase in CD62L^−^CD44^+^ T_reg_ cells and a reduction in CD62L^+^CD44^−^ T_reg_ cells; no difference was found in IL-10, IFNγ or IL-17 expression (Supplementary Fig. 1j–o). We next tested whether the coinhibitory receptors found in effector T_reg_ cells could be attributed to an intrinsic or extrinsic requirement of PD-1. For this analysis, we generated *Pd1*^*fl/fl-DTR-Tdtomato*^ (*Pd1*^*fl/fl*^) mice, in which *loxP* sites were inserted either side of exons 2 and 3 of the *Pd1* gene^25^. In addition, an inserted IRES drove expression of the tdTomato reporter and the diphtheria toxin receptor (DTR). We crossed this strain to *Foxp3*^*ERT2-Cre-eGFP*^ (ref. ^26^) to generate *Pd1*^*fl/fl-DTR-Tdtomato*^*Foxp3*^*ERT2-Cre-eGFP*^ (*Pd1*^*fl/fl*^*Foxp3*^*ERT2Cre*^) mice (Supplementary Fig. 2a–d). Using these animals, we repeated experiments after tamoxifen treatment. We observed an increase in FoxP3 expression (Fig. 1m,n) but no change in CD25, Helios, Tbet, Gata3 or RORγt expression or in the frequency of CD62L^−^CD44^+^ T_reg_ cells or CD62L^+^CD44^−^ T_reg_ cells (Supplementary Fig. 2e–k). Consistent with our findings in *Pd1*^*−/−*^*Foxp3*^*RFP*^ mice, only CD30 showed significantly increased expression in *Pd1*^*fl/fl*^*Foxp3*^*ERT2Cre*^ T_reg_ cells; no changes were noted in CTLA4, GARP, GITR or TIGIT (Fig. 1o–p and Supplementary Fig. 2l–o). Again, a compensatory increase in the coinhibitory receptor network was noted (Fig. 1q,r and Supplementary Table 2). By contrast, no change in cytokine expression was observed (Supplementary Fig. 2p–r). We also found significant upregulation of *Tnfrsf8* (CD30) mRNA (Fig. 1s,t). Finally, we tested whether PD-1 intrinsically controlled CD30. Using *Pd1*^*fl/fl-DTR-Tdtomato*^ *Foxp3*^*ERT2-Crehet-eGFP*^ (*Pd1*^*fl/fl*^*Foxp3*^*ERT2Cre+/−*^) mice, we found that CD30 was intrinsically inhibited by PD-1 in T_reg_ cells (Fig. 1u–w). No change in GARP or TIGIT expression was noted, but there was intrinsic upregulation of GITR and CTLA4 (Supplementary Fig. 2s–v, right panel).

### PD-1 deficiency does not alter proliferation or apoptosis of T_reg_ cells

In ex vivo splenocyte experiments, we found higher proportions of Ki67^+^ expression in both *Pd1*^*−/−*^ T_reg_ cells and CD4^+^FoxP3^*−*^ T cells compared with WT cells; however, contrary to previous reports^9,11^, these differences were not significant (Supplementary Fig. 3a–c). WT and *Pd1*^*−/−*^ T_reg_ cells stimulated with αCD3, αCD28 and IL-2 also showed no difference in proliferation (Supplementary Fig. 3d,e). T_reg_ cells expressing CD30, CTLA4, GITR and TIGIT were highly proliferative in both WT and *Pd1*^*−/−*^ animals (Supplementary Fig. 3f,g). No change in Annexin V staining or total live cells was observed between WT and *Pd1*^*−/−*^ T_reg_ cell populations (Supplementary Fig. 3h,i). A similar profile was observed for CD4^+^FoxP3^*−*^ T cells (Supplementary Fig. 3j,k). No change in Bcl2 expression was noted in either T_reg_ cells or CD4^+^FoxP3^*−*^ T cells (Supplementary Fig. 3l,m) between the WT and *Pd1*^*−/−*^ cohort. On further analysis of the FoxP3 compartment, WT T_reg_ cells expressing the CD30 network of coinhibitory receptors showed a significantly higher apoptotic index, whereas no such change was observed in *Pd1*^*−/−*^CD30^+^ T_reg_ cells (Supplementary Fig. 3n,o).

### CD30 can enhance *Pd1*^*−/−*^ T_reg_ function within the TME

**Fig. 2:**
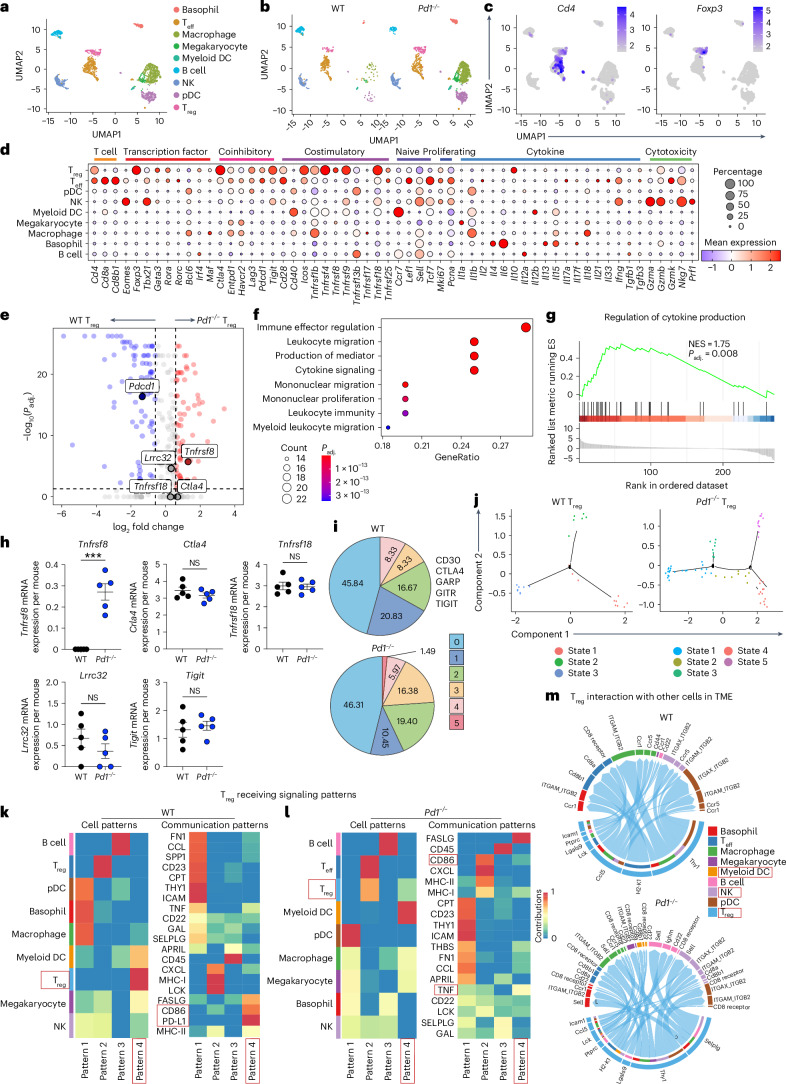
scRNA-seq analysis identifies CD30 expression in *Pd1*^*−/−*^ T_reg_ cells in the TME. WT and *Pd1*^*−/−*^ mice TILs were harvested on day 14 and subjected to scRNA-seq. **a**, UMAP view of 1,810 enriched TILs color-coded by assigned cell type. **b**, WT and *Pd1*^*−/−*^ TILs, decoupled to show similar clustering between the samples. **c**, UMAP view of *CD4* and *Foxp3* expression. **d**, Dot plot of candidate gene expression in immune clusters. **e**, Differential gene expression analysis of *Pd1*^*−/−*^ T_reg_ cells versus WT T_reg_ cells. **f**, GO enrichment analysis identifying the functions of upregulated genes in *Pd1*^*−/−*^ T_reg_ cells. **g**, GSEA showing enhanced regulation of cytokine production of *Pd1*^*−/−*^ T_reg_ cells. NES, normalized enrichment score. **h**, mRNA expression of *Tnfrsf8*, *Ctla4*, *Tnfrsf18* (GITR), *Lrrc32* (GARP) and *Tigit* transcripts in WT and *Pd1*^*−/−*^ T_reg_ cells in individual animals. **i**, Average percentages of T_reg_ cells expressing 0–5 *Tnfrsf8* (CD30), *Ctla4*, *Tnfrsf18* (GITR), *Lrrc32* (GARP) and *Tigit* transcripts in WT and *Pd1*^*−/−*^ T_reg_ cells in the TME. **j**, Pseudotime trajectory analysis of WT (left) and *Pd1*^*−/−*^ T_reg_ (right) cells. **k**,**l**, Heatmap showing cell patterns and received signaling patterns of WT (**k**) and *Pd1*^*−/−*^ (**l**) TILs. **m**, Chord diagram visualizing numbers of interactions between T_reg_ cells and TILs in WT (top) and *Pd1*^*−/−*^ (bottom) cohorts. The inner bar size is proportional to the signal strength received by the TILs from T_reg_ cells. Statistical analyses were performed using two-tailed Wilcoxon rank-sum test with Bonferroni correction in **e**, Fisher’s exact test with FDR correction in **f**, two-tailed Kolmogorov–Smirnov test with FDR correction in **g**, and two-tailed unpaired Student’s *t*-test in **h**. Data are mean ± s.e.m. from *n* = 5 mice per cohort in **h**. ***P < 0.001. For the results shown in **k**–**m**, CellChat was used to model communication probabilities based on the law of mass action and identify significant communications using permutation tests. *P*_adj__._, adjusted *P* value.

We established B16 tumors in WT and *Pd1*^*−/−*^ mice. Tumor growth was monitored (Supplementary Fig. 4a), and tumor-infiltrating lymphocytes (TILs) were harvested and subjected to scRNA-seq analysis. We used germline *Pd1*^*−/−*^ mice to generate a resource that would be relevant to the clinical setting. Immune cell clusters in WT and *Pd1*^*−/−*^ TILs were defined (Fig. 2a–d); then, T_reg_ cell clusters were determined based on *Cd4* and *Foxp3* (Fig. 2c and Supplementary Table 3a). Clusters were visualized with uniform manifold approximation and projection (UMAP) following Louvain clustering. Differential gene expression analysis, which was illustrated using a volcano plot, showed that *Tnfrsf8* was significantly upregulated in *Pd1*^*−/−*^ T_reg_ cell TILs compared with other canonical coinhibitory receptors (Fig. 2e and Supplementary Table 3b). Among effector T (T_eff_) cells, we found an increase in *Gzm* transcripts in *Pd1*^*−/−*^ TILs compared with WT TILs (Supplementary Fig. 4c,d and Supplementary Table 3c,d). Gene ontology (GO) enrichment analysis and gene set enrichment analysis (GSEA) demonstrated that the *Pd1*^*−/−*^ T_reg_ cell cohorts had enhanced cytokine signaling (Fig. 2f,g and Supplementary Table 3e,f). We interrogated whether the changes in the coinhibitory network in steady state in *Pd1*^*−/−*^ T_reg_ cells compared with WT T_reg_ cells was preserved in the TME. There was a significant increase in *Tnfrsf8* expression in *Pd1*^*−/−*^ T_reg_ cells compared with WT T_reg_ cells (Fig. 2h and Supplementary Fig. 4b). Using Boolean analysis, we found that the altered coinhibitory receptor network observed in steady-state *Pd1*^*−/−*^ T_reg_ cells was preserved at the transcript level in *Pd1*^*−/−*^ T_reg_ cell TILs (Fig. 2i).

Pseudotime and trajectory analysis using the Monocle plugin in SeqGeq demonstrated that WT and *Pd1*^*−/−*^ T_reg_ cells combined had seven states of differentiation (Supplementary Fig. 4e,g). When WT and *Pd1*^*−/−*^ T_reg_ cells were separated, pseudotime trajectory analysis revealed five differentiation states in *Pd1*^*−/−*^ T_reg_ cells and three in WT T_reg_ cell TILs (Fig. 2j and Supplementary Table 3g,h). WT T_reg_ cells showed a tissue-resident state marked by *St2* (state 1), an immunosuppressive phenotype marked by *Tigit* (state 2) and a cytotoxic state (state 3). By contrast, *Pd1*^*−/−*^ T_reg_ cells comprised a type 1 phenotype characterized by either IFNγR (state 1) or *Stat1* expression (state 3), a cytotoxic phenotype (state 2), an immune-regulatory phenotype (state 4) and a TNFR-enriched differentiation program (state 5).

Using CellChat analysis, we determined the communication program of T_reg_ cells within the TME. Whereas WT T_reg_ cells showed a single incoming pattern dominated by CD86 (activating CTLA4) and PD-L1 (through PD-1), *Pd1*^*−/−*^ T_reg_ cells showed dual incoming signaling patterns dominated by CD86 and TNF (activating TNF receptors through CD30; red boxes in Fig. 2k,l). We next investigated the signals contributed by all immune cells among the TILs; we found no significant difference in incoming signaling patterns between the WT and *Pd1*^*−/−*^ cohorts, but *Pd1*^*−/−*^ natural killer (NK) cells contributed less to outgoing signaling patterns (Supplementary Fig. 5a,b). We next determined the crosstalk of T_reg_ cells with other immune cells within the TME. Analysis of receptor–ligand interactions, the results of which were visualized using a chord diagram, showed increases in expression of several binding partners of *Pd1*^*−/−*^ T_reg_ cells with NK cells and myeloid dendritic cells (DCs) (Fig. 2m) compared with WT T_reg_ cells. In turn, communication of NK cells with T_eff_ cells was predicted to be diminished, whereas no changes in myeloid DC communication were noted (Supplementary Fig. 5c,d). This predictive analysis suggests that the interaction between *Pd1*^*−/−*^ T_reg_ cells and NK cells may influence the interactions of NK cells with T_eff_ cells within the TME; however this would need to be validated in future studies and is beyond the scope of the current work.

### Functional validation of scRNA-seq and CellChat analysis of the TME

**Fig. 3:**
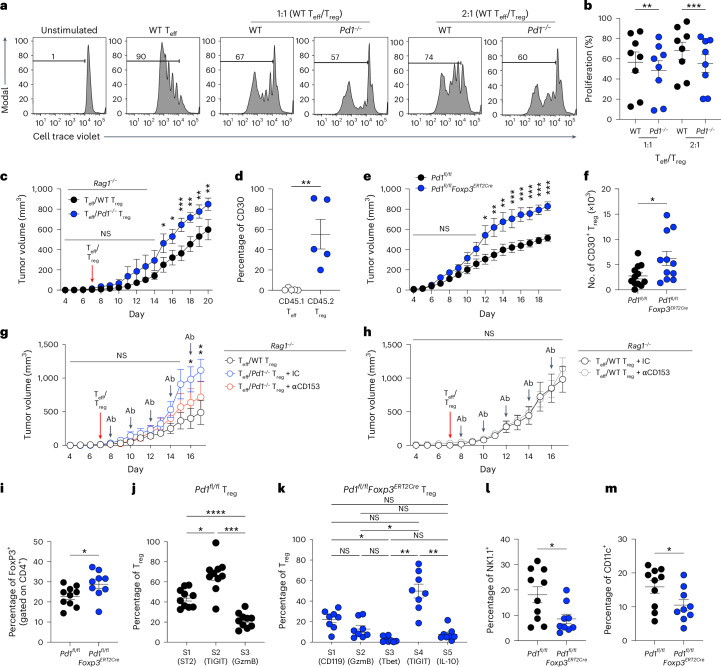
PD-1 deficiency enhances T_reg_ cell function through CD30 within the TME. **a**,**b**, Suppressive function of T_reg_ cells from *Foxp3*^*RFP*^ and *Pd1*^*−/−*^*Foxp3*^*RFP*^ mice (*n* = 8 per group) as demonstrated by a representative CellTrace Violet flow plot (**a**) and percentages of T_eff_ cell proliferation (**b**). **c**,**d**, For *Rag1*^*−/−*^ mice with B16F10 melanoma cells and reconstituted with CD45.1^+^ T cells and CD45.2^+^ T_reg_ cells from either WT or *Pd1*^*−/−*^ mice, tumor volumes (*n* = 8 for WT and *n* = 6 for *Pd1*^*−/−*^ groups) (**c**) and frequencies of CD30 expression on CD45.1^+^ T_eff_ and CD45.2^+^ *Pd1*^*−/−*^ T_reg_ cells in TILs (*n* = 5) (**d**) are shown. **e**,**f**, For tamoxifen-treated *Pd1*^*fl/fl*^ and *Pd1*^*fl/fl*^*Foxp3*^*ERT2Cre*^ mice with tumors, tumor volumes (*n* = 10 for *Pd1*^*fl/fl*^ and *n* = 9 for *Pd1*^*fl/fl*^*Foxp3*^*ERT2Cre*^) (**e**) and absolute counts of CD30^+^ T_reg_ cells (*n* = 12 for *Pd1*^*fl/fl*^ and *n* = 11 for *Pd1*^*fl/fl*^*Foxp3*^*ERT2Cre*^) (**f**) are shown. **g**, Tumor-bearing *Rag1*^*−/−*^ mice were reconstituted with different T cell populations (*n* = 3 for T_eff_ plus WT T_reg_ cell group and *n* = 7 for T_eff_ plus *Pd1*^*−*^^*/*^^*−*^ Treg cell groups); in the Teff plus *Pd1*^*−/−*^ Treg cohorts, mice were treated with either 0.1 mg anti-IgG2a (*n* = 5) or anti-CD153 (CD30L, *n* = 4), and tumors were measured. **h**, Tumor-bearing *Rag1*^*−/−*^ mice were reconstituted with T_eff_ plus WT T_reg_ (treated with either 0.1 mg anti-IgG2a (*n* = 5) or anti-CD153 (*n* = 5)), and tumors were measured. Ab, antibody. **i**–**m**, Tamoxifen-treated and tumor-bearing *Pd1*^*fl/fl*^ (*n* = 10) and *Pd1*^*fl/fl*^*Foxp3*^*ERT2Cre*^ (*n* = 8 or 9) mice were immunophenotyped on day 19: frequencies of FoxP3 (**i**); ST2, TIGIT and GzmB in *Pd1*^*fl/fl*^ mice (**j**); IFNγR (CD119), GzmB, Tbet, TIGIT and IL-10 in *Pd1*^*fl/fl*^*Foxp3*^*ERT2Cre*^ mice (**k**); NK cells (**l**); and DCs (**m**) are shown. Data are presented as the mean ± s.e.m. Each data point represents an in vitro biological replicate in **b** or an individual animal in **f**, **i**, **l** and **m**. Data points in each group were matched to the same individual mouse in **d**, **j** and **k**. One-way analysis of variance (ANOVA) with Sidak’s multiple comparison was used in **b**; two-way ANOVA with Sidak’s multiple comparison in **c**, **e**, **g** and **h**, two-tailed paired Student’s *t*-test in **d**; two-tailed unpaired Student’s *t*-test in f, **i**, **l** and **m**; and matched one-way ANOVA with Sidak’s multiple comparison in **j** and **k**. Cumulative data from *n* = 3 independent experiments are shown. *P < 0.05, **P < 0.01, ***P < 0.001, ****P < 0.0001.

Similar to previous work, we found that *Pd1*^*−/−*^ T_reg_ cells were significantly more suppressive in vitro^27^ (Fig. 3a,b). For analysis of in vivo suppressive function, *Rag1*^*−/−*^ mice bearing tumors were reconstituted with either WT or *Pd1*^*−/−*^ CD45.2^+^ T_reg_ cells along with WT CD45.1^+^ naive CD4^+^ T cells. The *Rag1*^*−/−*^ cohort reconstituted with *Pd1*^*−/−*^ T_reg_ cells showed significantly enhanced tumor growth compared with those reconstituted with WT T_reg_ cells (Fig. 3c). No significant differences in T_reg_ cell frequency, cytokine production or cytokine production in CD4^+^ T_eff_ cells were observed between the two groups (Supplementary Fig. 6a–d). We next sought to confirm the results of our scRNA-seq and CellChat analysis of CD30 expression in *Pd1*^*−/−*^ T_reg_ cells. Using the same *Rag1*^−/−^ tumor model, we found that CD30 frequency was significantly increased in T_reg_ cells but not in CD45.1^+^ T_eff_ cells among TILs (Fig. 3d). We next tested whether T_reg_ cells in *Pd1*^*fl/fl*^*Foxp3*^*ERT2Cre*^ mice had a tumor-protective role similar to that of global *Pd1*^*−/−*^ T_reg_ cells. For this purpose, we established tumors in control mice treated with phosphate-buffered saline (PBS) and *Pd1*^*fl/fl*^*Foxp3*^*ERT2Cre*^ mice after tamoxifen treatment. Significantly increased tumor growth was noted in the cohort with PD-1-deficient T_reg_ cells (Fig. 3e), along with significant increases in absolute numbers of CD30^+^ TIL T_reg_ cells in *Pd1*^*fl/fl*^*Foxp3*^*ERT2Cre*^ mice compared with *Pd1*^*fl/*fl^ control mice (Fig. 3f). By contrast, we found no change in absolute numbers of FoxP3^+^, IFNγ^+^, IL-10^+^, GITR^+^ or CTLA4^+^ TIL T_reg_ cells (Supplementary Fig. 6e–i).

Next, *Rag1*^*−/−*^ mice were reconstituted with either WT or *Pd1*^*−/−*^ CD45.2^+^ T_reg_ cells along with WT CD45.1^+^ naive CD4^+^ T cells, but animals receiving *Pd1*^*−/−*^ CD45.2^+^ T_reg_ cells were injected with either an isotype control or anti-CD30L (also known as anti-CD153). We found that blocking the CD30 pathway with anti-CD30L significantly inhibited tumor growth after 14 days in the *Pd1*^*−/−*^ CD45.2^+^ T_reg_ cell cohort compared with animals that received the isotype control. This decrease in tumor growth caused by anti-CD153 treatment reversed most of the increase observed in animals reconstituted with *Pd1*^*−/−*^ CD45.2^+^ T_reg_ cells compared with those reconstituted with WT T_reg_ cells (Fig. 3g). Next, we tested whether anti-CD153 treatment also had an impact on the protumor function of WT T_reg_ cells. In *Rag1*^*−/−*^ animals reconstituted with B16 melanoma, WT CD45.2^+^ T_reg_ cells and CD45.1^+^ naive CD4^+^ T cells, we found no difference in tumor growth between those in which reconstitution was followed by anti-CD153 treatment and those who received the isotype control (Fig. 3h). These data indicate that the CD30/CD30L pathway is a specific target for PD-1-deficient T_reg_ cell function and plays a minimal part in WT T_reg_ cell function. Immunophenotyping data showed that anti-CD153 treatment did not deplete the frequency of either T_reg_ cells or T_eff_ cells in any condition, in contrast to previous reports^28^ (Supplementary Fig. 6j–m). We next sought to confirm our Monocle-predicted differentiation states and the results of our CellChat analysis. *Pd1*^*fl/fl*^*Foxp3*^*ERT2Cre*^ mice were reconstituted with tumors after tamoxifen treatment, and T_reg_ cells were characterized. We found a significant increase in FoxP3 expression among the TILs (Fig. 3i). We then subcharacterized T_reg_ cells based on our pseudotime trajectory analysis and detected the presence of all three substates in the WT and five different substates in the PD-1-deficient T_reg_ cell TILs (Fig. 3j,k). Next, we tested whether PD-1 deficiency in T_reg_ cells affected NK and DCs among TILs per the predictive CellChat analysis. We observed significant decreases in NK and DC frequencies among the TILs (Fig. 3l,m) but no changes for other functional markers (Supplementary Fig. 6n–t).

### CD30 expression is driven by STAT5 signaling in *Pd1*^*−/−*^ T_reg_ cells

**Fig. 4:**
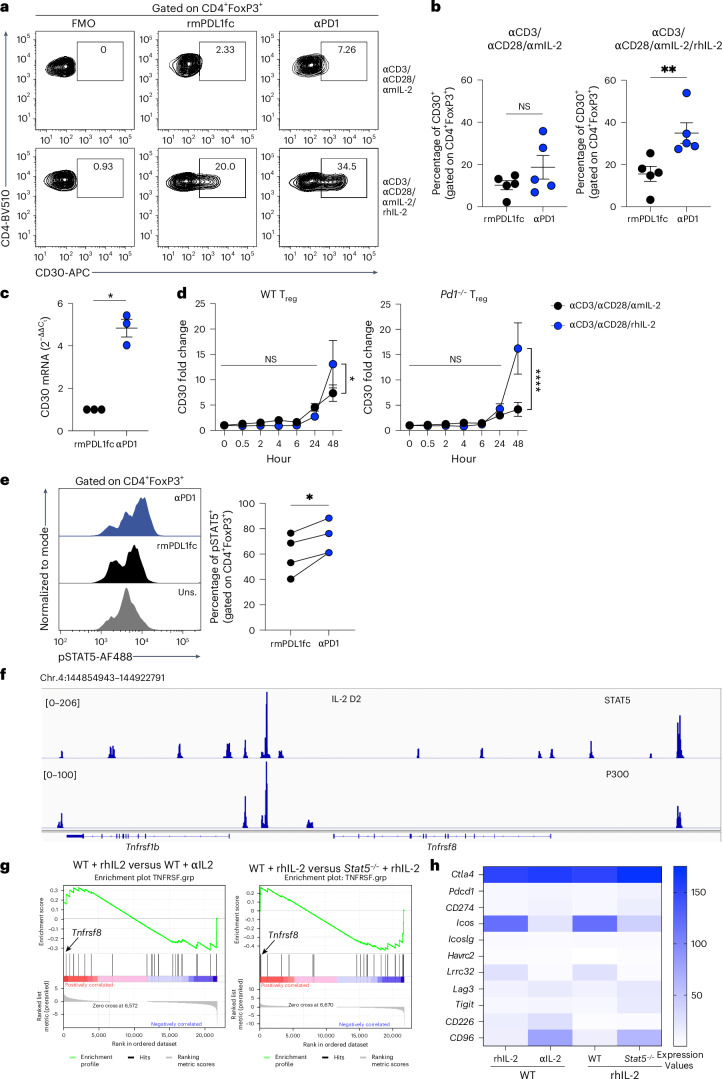
PD-1 regulates CD30 expression via IL-2–STAT5 signaling. **a**–**c**, WT T_reg_ cells from C57BL/6 mice were stimulated with αCD3 and αCD28 and cultured with either anti-mouse IL-2 (αmIL-2) or αmIL-2 with recombinant human IL-2 (rhIL-2). Cells were either cultured in recombinant mouse PD-L1 Fc (rmPD-L1-Fc)-coated well plates or with αPD-1 to block the PD-1–PD-L1 interaction: a representative plot of CD30 expression (**a**), a summary of CD30 expression in five independent experiments (**b**) and CD30 mRNA expression in three independent experiments (**c**) are shown. **d**, T_reg_ cells from C57BL/6 (WT) and *Pd1*^*−/−*^ mice were stimulated with αCD3 and αCD28 and cultured with either αmIL-2 or rhIL-2. At the indicated time points, CD30 expression on T_reg_ cells was plotted as the fold change in expression compared with the unstimulated condition (0 h). Data from seven independent experiments are shown. **e**, WT T_reg_ cells were stimulated with αCD3, αCD28 and αIL-2 in a PD-L1 Fc-coated plate. αPD-1 was added to block the PD-1–PD-L1 interaction. After 48 h, cells were stimulated with IL-2 for 15 min, and phospho-STAT5 (pSTAT5) was measured; the results are shown on the left. A summary of pSTAT5 in CD30^+^ T_reg_ cells is shown on the right. Uns., unstimulated. **f**, Representative ChIP–seq tracks and peaks detected by STAT5 (upper) and p300 (lower) at the *Tnfrsf8* gene locus of naive WT CD4^+^ cells stimulated with αCD3, αCD28 and rhIL-2 for 3 days. **g**,**h**, Naive WT and *Stat5*^*−/−*^ CD4^+^ cells were stimulated with αCD3, αCD28 in the presence of αIL-2 for 72 h, washed and restimulated with αCD3 along with either αIL-2 or rhIL-2 for 4 days: GSEA plots showing *Tnfrsf* family gene rankings in IL-2-treated WT CD4^+^ cells compared with αIL-2-treated WT CD4^+^ cells (left) and *Stat5*^*−/−*^ CD4^+^ cells (right) (**g**); and heatmaps showing expression of coreceptors and ligands in WT and *Stat5*^*−/−*^ CD4^+^ cells (**h**) are shown. Data are presented as the mean ± s.e.m.; each data point represents an independent experiment. Statistical analyses were performed using two-tailed paired Student’s *t*-tests (**b**, **c** and **e**) or two-way ANOVA with Sidak’s multiple comparison (**d**). *P < 0.05, **P < 0.01, ****P < 0.0001.

We measured the ability of PD-1 to inhibit anti-CD3, anti-CD28 stimulation and IL-2-induced expression of CD30 in WT FoxP3^+^ T cells. In the absence of IL-2, neither PD-1 inhibition nor activation using a PD-L1 Fc chimera altered CD30 expression. In the presence of IL-2, PD-1 inhibition led to significant increases in both protein and mRNA CD30 expression compared with the PD-L1 Fc condition (Fig. 4a–c). Similarly, we observed a significant increase in CD30 expression in both WT and *Pd1*^*−/−*^ T_reg_ cells stimulated with anti-CD3/28 in the presence of IL-2 (Fig. 4d).

We next found that PD-1 activation through PD-L1 downregulated STAT5, whereas anti-PD-1 enhanced STAT5 phosphorylation in T_reg_ cells (Fig. 4e). Using previously published RNA-seq and CHIP–seq (chromatin immunoprecipitation followed by sequencing) datasets, we explored the effects of STAT5 on CD30 expression^29^. We found that STAT5 bound to the *Tnfrsf8* gene locus in WT CD4^+^ T lymphoblasts stimulated with IL-2 for 48 h and shared two sites with p300 on either side of the gene (Fig. 4f). To confirm that STAT5 positively regulated CD30 expression, we performed GSEA, comparing WT CD4^+^ T cells subjected to IL-2 stimulation with the anti-IL-2 condition, and IL-2-stimulated WT CD4^+^ T cells with *Stat5*^*−/−*^ CD4^+^ T cells. Genes were then ranked with respect to their differential expression between the comparison groups; we found that the gene encoding CD30, a member of the TNFRSF family, was highly ranked among the genes induced by IL-2 in the presence of STAT5 (Fig. 4g). By contrast, the TNFRSF family as a group was not significantly enriched in either condition. Next, we evaluated whether a similar pattern in fold change could be observed for other common coinhibitory receptors and found that STAT5 did not significantly regulate clinically relevant coinhibitory receptors (Fig. 4h).

### Spatial transcriptomic analysis of *Pd1*^*−/−*^ T_reg_ cell interactions in the TME

**Fig. 5:**
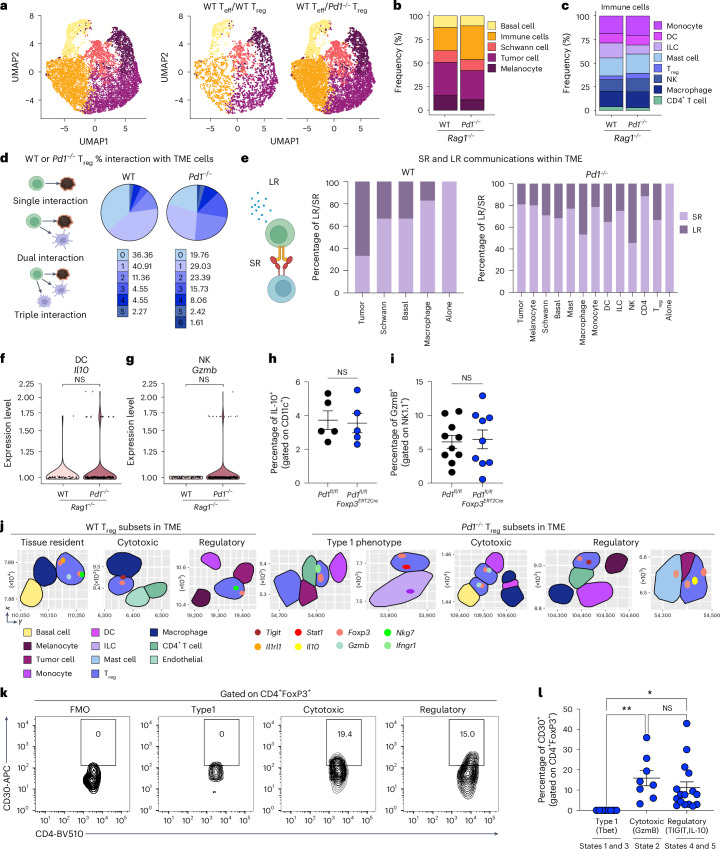
Spatial transcriptomics of *Pd1*^*−/−*^ T_reg_ cell communication programs in TME. **a**–**g**, Tumor-bearing *Rag1*^*−/−*^ mice were reconstituted with different T cell subsets and then subjected to CosMx spatial transcriptomics: UMAPs of 9,231 cells derived from WT T_eff_/WT T_reg_ cell and WT T_eff_/*Pd1*^*−/−*^ T_reg_ cell cohorts, color-coded by assigned cell type (left), and WT and *Pd1*^*−/−*^ T_reg_ cell cohorts decoupled (middle and right) (**a**); frequencies of all cells in the TME (**b**); immune cells subclusters and frequencies of immune cell subsets among WT and *Pd1*^*−/−*^ T_reg_ cells (**c**); a graphical representation of cell–cell interaction analysis (left) and numbers of cell–cell interactions of WT T_reg_ cells (middle) and *Pd1*^*−/−*^ T_reg_ cells (right) (**d**); a graphical representation of SR and LR gene definition by manual annotation (left), and frequencies of LR and SR interactions of WT (middle) and *Pd1*^*−/−*^ T_reg_ cells (right) (**e**); and DC *Il10* expression (**f**) and *Gzmb* expression in NK cells (**g**) according to clustered Wilcoxon rank-sum test are shown. **h**,**i**, Tamoxifen-treated, tumor-bearing *Pd1*^*fl/fl*^ and *Pd1*^*fl/fl*^*Foxp3*^*ERT2Cre*^ mice were immunophenotyped on day 19: the frequencies of IL-10 expression in TIL DCs (*n* = 5 per group) (**h**) and GzmB expression in TIL NK cells (*n* = 10 for *Pd1*^*fl/fl*^ and *n* = 9 for *Pd1*^*fl/fl*^*Foxp3*^*ERT2Cre*^) (**i**) are shown. **j**, For mice as in **a**–**g**, FOVs showing colocalization of different WT and *Pd1*^*−/−*^ T_reg_ cell states and their interactions within the TME. **k**,**l**, Tamoxifen-treated, tumor-bearing *Pd1*^*fl/fl*^*Foxp3*^*ERT2Cre*^ mice were immunophenotyped (*n* = 8): representative flow cytometry results (**k**) and a summary of CD30 expression in T_reg_ cell subsets (**l**) are shown. Data represent individual cells in **f** and **g**. In **h**, **i** and **l**, data are from three independent experiments, and the mean ± s.e.m. is shown. In **h** and **i**, each data point represents an individual mouse. In **l**, data points are matched within animals. Two-tailed clustered Wilcoxon rank-sum test was used for f and g, two-tailed unpaired Student’s *t*-test for **h** and **i**, and one-way ANOVA with Sidak’s multiple comparison test for **l**. *P < 0.05, **P < 0.01. Illustrations in **d** and **e** created using BioRender.com. ILC, innate lymphoid cell.

To visualize the predicted CellChat interactions and pseudotime trajectory analysis, we performed CosMx spatial transcriptomics. *Rag1*^*−/−*^ mice engrafted with tumors were reconstituted with either WT or *Pd1*^*−/−*^CD45.2^+^ T_reg_ cells, along with WT CD45.1^+^ naive CD4^+^ T cells. Tumor sections stained with hematoxylin and eosin (H&E) from both groups of animals were evaluated, and identical tissues were used for the CosMx analysis. Fields of view (FOVs) that were enriched for both immune infiltration and surrounding tumor tissue were chosen for analysis. A combination of cell segmentation analysis and immune cell profiling enabled us to identify single cells and their corresponding cell types within the tissue (Supplementary Fig. 7a,b). Immune cell profiling was performed using Louvain clustering analysis across all FOVs from the tested samples, integrating RNA transcripts for enhanced resolution (Fig. 5a, Supplementary Fig. 7c and Supplementary Table 4a). This approach allowed subdivision and classification of the ‘immune cells’ cluster based on specific immune cell markers, which were then mapped to single cells within the tissue (Supplementary Fig. 7b, right). Frequencies of the various cell populations in the two cohorts were analyzed (Fig. 5b), with the ‘immune cells’ cluster further subdivided by specific markers (Fig. 5c). Pathway analysis indicated upregulation of leukocyte migration pathways in *Pd1*^*−/−*^ T_reg_ cells compared with WT T_reg_ cells (Supplementary Fig. 7d). The CosMx dataset was then used for several descriptive visual analyses of T_reg_ cells in the TME. First, all T_reg_ cell IDs and cell coordinates were obtained from the FOV object (Supplementary Table 4b). Each T_reg_ cell interaction with neighboring cells was assigned as an event, and the number of events was determined through manual annotation of the FOV images. These interaction events were classified as single, dual or multiple (Fig. 5d). T_reg_ cells were grouped based on their interactions with respective tumor or immune cells as follows. All the T_reg_ cells interacting with a specific cell type were grouped using their unique cell ID (Supplementary Table 4c). Enriched transcripts in each group were determined using differential gene expression analysis to identify the short-range (SR) and long-range (LR) communication signals expressed by T_reg_ cells (Supplementary Table 4d,e). We found that T_reg_ cells used combinations of SR signals (ligand or receptor expression) to interact with CD4^+^ T_eff_ cells, NK cells, DCs and macrophages (Fig. 5e), together with tissue-resident cells within the TME. However, it should be noted these were descriptive analyses using manual annotation that suggested possible interactions with other immune cells. We found potential interactions of T_reg_ cells with NK and DCs using either SR or LR communication signals. Hence, we analyzed the mRNA of DCs and NK cells for functional changes. Although we observed slight increases in *Il10* and *Gzmb* expression in DCs and NK cells (Fig. 5f,g), when we attempted to validate these results in vivo using tumor-bearing *Pd1*^*fl/fl*^ and *Pd1*^*fl/fl*^*Foxp3*^*ERT2Cre*^ mice, no difference in IL-10 or GzmB protein expression in DCs or NK cells was found (Fig. 5h,i).

As our CosMx panel did not include *Tnfrsf8*, we investigated whether we could identify which cell type within the TME predominantly communicated through *Tnfsf8* (CD30L) with T_reg_ cells, as CD30 is the only binding partner for CD30L. We found CD30L expression in tissue-resident cells, tumor cells and immune cells in the cohorts treated with WT and *Pd1*^*−/−*^ T_reg_ cells (Supplementary Fig. 7e and Supplementary Table 4f). We next visualized cells expressing CD30L that were in contact with T_reg_ cells across all the FOVs. The frequency of each cell type was visualized, and the possible interactions of all cells with T_reg_ cells via CD30L within the TME were identified (Supplementary Fig. 7f). The mRNA data suggested that CD30L was ubiquitously expressed in all cell types in the TME.

We next sought to visually validate our pseudotime trajectory analysis in the TME and found three different subsets of T_reg_ cells in the WT T_reg_ cell-treated cohorts and five different subsets in the *Pd1*^*−/−*^ T_reg_ cell-treated cohort (Fig.5j). Taken together, the results of our descriptive analysis of the CosMx data indicated the presence of CD30L in all cells in the TME and identified different T_reg_ cell subsets in the TME. Finally, we validated the expression of CD30 in these T_reg_ cell subsets in the TME in *Pd1*^*fl/fl*^*Foxp3*^*ERT2Cre*^ mice. We observed CD30 expression in the regulatory and cytotoxic T_reg_ cell subsets (Fig. 5k,l). Hence, our validation studies suggest that it is the regulatory subsets of the *Pd1*^*−/−*^ T_reg_ cell population in the TME that express CD30, and that blocking CD30/CD30L inhibits this regulatory axis.

### PD-1 disruption not the TME enhances CD30 expression in human T_reg_ cells

**Fig. 6:**
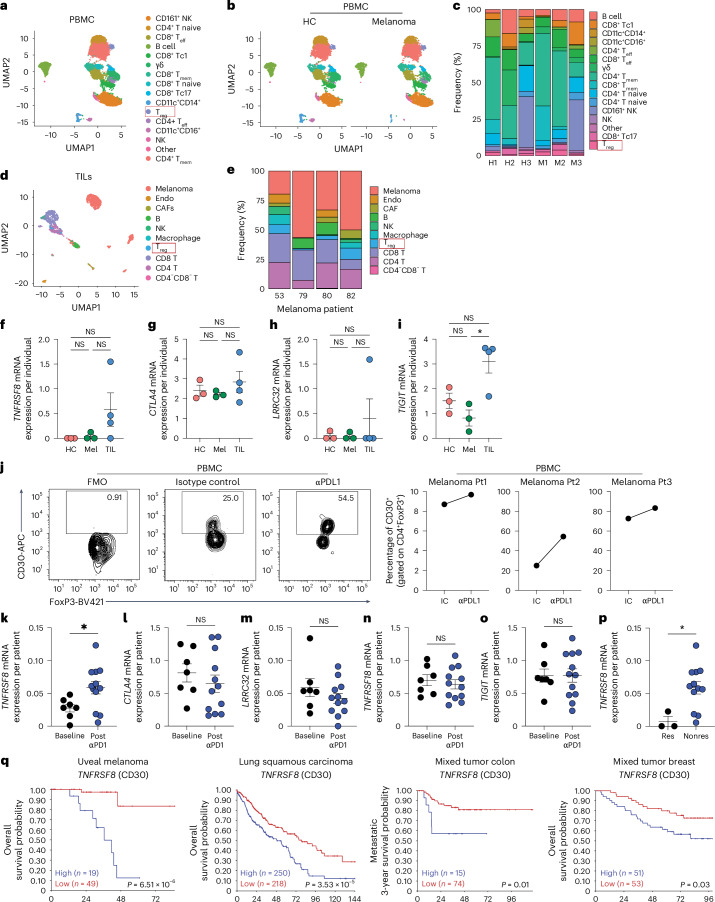
Anti-PD-1 and not the melanoma TME enhances CD30 expression by human T_reg_ cells. **a**–**c**, PBMCs from HC and stage IV melanoma patients (Mel) were subjected to scRNA-seq: a UMAP view of 11,634 cells, with all cells colored by cell type (**a**); HC and Mel PBMC UMAPs showing homogeneity of clusters between the two cohorts (**b**); and frequencies of each cluster within individual samples (**c**) are shown. H, healthy; M, melanoma. **d**,**e**, Publicly available TIL and tumor cell data were mined and analyzed: a UMAP view of 1603 cells, with all cells colored by cell type (**d**), and frequencies of each cluster in individual patients (**e**) are shown. Endo, endothelial. **f**–**i**, mRNA expression of *TNFRSF8* (**f**), *CTLA4* (**g**), *TIGIT* (**h**) and *LRRC32* (GARP) (**i**) in T_reg_ cells from PBMCs of HC individuals (*n* = 3) and Mel patients (*n* = 3), and T_reg_ cells from TILs (*n* = 4), where each data point represents an individual. **j**, HC and Mel PBMCs were cultured with isotype control (IC) or anti-PD-L1 antibody (αPD-L1): a representative flow plot of CD30 expression in T_reg_ cells (left) and a summary of CD30 expression in T_reg_ cells (right) are shown. Pt, patient. **k**–**o**, mRNA expression of *TNFRSF8* (**k**), *CTLA4* (**l**), *GARP* (**m**), *TNFRSF18* (**n**) and *TIGIT* (**o**) in T_reg_ cells from individual patients at baseline and after anti-PD-1 treatment. **p**, *TNFRSF8* mRNA expression in T_reg_ cells in each patient identified as a responder (Res) or nonresponder (Nonres) to anti-PD-1 immunotherapy. **q**, Kaplan–Meier analysis of overall survival of patients diagnosed with different cancers corresponding to CD30 expression, with *P* values indicated. Data are presented as the mean ± s.e.m., with each data point representing an individual from at least three independent experiments in **f**–**i** and **k**–**p**. Statistical analysis was performed using one-way ANOVA with multiple Sidak’s comparison (**f**–**i**) or two-tailed unpaired Student’s *t*-test (**k**–**p**). *P < 0.05. The survival curve statistical analysis for **q** was performed using a two-tailed log-rank test with the R2 Genomics Analysis and Visualization Platform.

We tested whether CD30 expression in human T_reg_ cells was driven by the TME or by anti-PD-1 effects by means of in-house BD Rhapsody scRNA-seq analysis of peripheral blood mononuclear cells (PBMCs) from stage IV human melanoma patients and healthy controls (HC). We also used a publicly available scRNA-seq dataset from melanoma TILs to validate our findings in naive melanoma TILs (Supplementary Table 5). Immune cell clusters in HC and melanoma samples (Fig. 6a–c) were defined using AbSeq protein expression and the top five gene transcripts expressed in each cluster (Supplementary Fig. 8a–c and Supplementary Table 6a,b), whereas the public dataset was analyzed using gene transcripts (Fig. 6d,e, Supplementary Fig. 9a and Supplementary Table 6c). We found no alteration of mRNA expression of CD30 or any other coreceptor in tumor-derived T_reg_ cells among either TILs or melanoma PBMCs compared with HC PBMCs (Fig. 6f–i and Supplementary Fig. 8d–g). Notably, expression of *TIGIT* was significantly enhanced when measured in individual patients, suggesting regulation of TIGIT by the TME. The results of this analysis suggested that the TME alone does not drive enhanced CD30 expression in T_reg_ cells among PBMCs or TILs in melanoma.

We next tested the effect of blocking the PD-1 pathway on CD30 expression in T_reg_ cells using in vitro cultures. T_reg_ cells cultured in the presence of an anti-PD-L1 antibody showed upregulation of CD30 for all three human melanoma patients (Fig. 6j), suggesting that it is not the TME but anti-PD-1 activity that drives CD30 expression in tumor T_reg_ cells.

We next analyzed the expression of CD30 in T_reg_ cells from a cohort of anti-PD-1-treated melanoma patients using a publicly available dataset. We first performed cluster analysis to classify immune cells (Supplementary Fig. 9b–e, and Supplementary Table 6d), followed by two types of analysis. In the first analysis, we tested CD30 expression and that of other coreceptors in patient samples both at baseline and after anti-PD-1 therapy. We found that when individual patient data were analyzed separately, *TNFRSF8* expression was significantly enhanced after anti-PD-1 therapy, whereas no changes were found for *CTLA4*, *LRRC32* (GARP), *TNFRSF18* (GITR) or *TIGIT* (Fig. 6k–o). The results of the single-cell analysis were consistent with these findings, with a significant difference in *TNFRSF8* expression and no differences for other coreceptors (Supplementary Fig. 9f–j). In the second analysis, we compared the expression of *TNFRSF8* in T_reg_ cells from responders and nonresponders to anti-PD-1 therapy. Again, we found that *TNFRSF8* was predominantly expressed within the nonresponder cohort, but no significant difference was noted for other coinhibitory receptors (Fig. 6p and Supplementary Fig. 9k–n). Finally, we tested whether CD30 could be used as a biomarker for prediction of cancer-free survival. We found that elevated CD30 expression in PBMCs was associated with poor survival in patients with uveal melanoma and those with breast, lung or colon cancer, indicating that CD30 could be used as a biomarker and therapeutic target that may determine disease outcomes after checkpoint therapy (Fig. 6q).

## Discussion

The use of antibodies against PD-1 and CTLA4 has been transformative in the management of many cancers, particularly melanoma. Despite the success of these treatments in certain cases, most cancer patients either fail to respond, relapse after an initial response or develop hyperprogressive disease in which PD-1 inhibition paradoxically stimulates tumor growth. The emerging literature suggests that anti-PD-1 immunotherapy can counterintuitively enhance T_reg_ cell numbers in a proportion of patients, driving resistance to immunotherapy. In this study, using clinically relevant global *Pd1*^*−/−*^ and biologically specific *Pd1*^*fl/fl*^ animals, we elucidated three key aspects of PD-1 biology in T_reg_ cells in steady state and in the TME. Specifically, we showed that PD-1 could inhibit CD30 coinhibitory receptor expression in (1) T_reg_ cells in steady state, (2) T_reg_ cells in the TME and (3) human T_reg_ cells in melanoma. These data provide insight into the complex relationship between PD-1 and T_reg_ cell function.

Our finding that *Pd1*^*−/−*^ T_reg_ cells were superior in function to WT T_reg_ cells was in line with recent reports on autoimmunity and infectious diseases^9,11^. This increase in function was not due to increased proliferation as measured in vitro or increased accumulation as measured in vivo. Hence, we explored an alternative hypothesis that an increase in T_reg_ cell function could result from compensatory upregulation of other well-known coinhibitory receptors such as GITR, CTLA4, TIGIT and GARP. However, the dominant coinhibitory receptor that was intrinsically regulated by PD-1 deficiency was CD30. This finding was unexpected but enabled us to identify a unique regulatory pathway in T_reg_ cells that could be manipulated to enhance checkpoint therapies. Indeed, the roles of CD30 in immunotherapy and T_reg_ cell biology have shown that CD30 plays a key part in T_reg_ cell function in graft versus host disease^30^. Hence, our data are complimentary to previous observations and are relevant to anti-PD-1 resistance, as patients who have enhanced T_reg_ cell numbers after PD-1 therapy could be treated with anti-CD30 antibodies. Murine studies have found that combining anti-CD30 therapy with other coreceptors can delete T_reg_ cells^28^; this finding was not corroborated in our system, in which we found that blocking CD30/CD30L preserved T_reg_ cell numbers within the TME.

The functional plasticity of T_reg_ cells has been well established, with important roles for cytokines^31^ and coreceptors^32^. PD-1 is vital for maintaining FoxP3 expression in both human^4^ and murine Tbet^+^ T_reg_ cells^6^. A recent study showed that *Pd1*^*−/−*^ T_reg_ cells were functionally plastic in the TME^8^ by acquiring an altered metabolic phenotype. This observation confirmed previous work by us and others, in which PD-1 deficiency was found to induce a glycolytic phenotype in T cells and innate immune cells (ILC2/ILC1)^33,34^. We found a similar phenotype in *Pd1*^*−/−*^ T_reg_ cells, with pseudotime analysis identifying type 1 differentiation states in T_reg_ cells. Therefore, we concluded that PD-1 does control the emergence of T_reg_ cells with type 1 phenotype; however, within the TME, it also controls T_reg_ cell subsets that can drive immune regulation through TNFRs, TIGIT and IL-10. We proposed that within the TME, several T_reg_ cell states would coexist, and their function would be determined by the predominant state during a particular space and time. We sought to confirm this using spatial transcriptomics to identify which T_reg_ cell states were associated with either immune cells or tumor cells. We found that T_reg_ cells expressing *Il10*, *Tigit*, and *Tnfrsf* were in proximity to tumor cells. Hence, *Pd1*^*−/−*^ T_reg_ cells could exhibit both functional plasticity and exert an immune suppressive function within the TME. Validation in mouse models further confirmed an increase in CD30 expression in the regulatory T_reg_ cell subsets within the TME.

The communication patterns of WT and *Pd1*^*−/−*^ T_reg_ cells in TME were of particular note. We showed that deleting PD-1 enhanced a second pattern of communication among T_reg_ cells that was primarily driven by TNF signaling, which may occur through TNFRs. Although GITR seemed to play a part in this communication pattern, it was CD30 that was predominantly activated. These data could potentially explain the lack of success of combination trials in which anti-PD-1 and GITR agonists have been used^35^. Similarly, anti-LAG3 combination trials have been proposed to overcome resistance; however, in our single-cell analysis, LAG3 was not altered in all T cell subsets. These data highlight the need for unsupervised analysis of T cells in clinical models of established large tumors as a proof of concept before building combination therapy trials using checkpoint antibodies.

Our data suggests that of all T_reg_ differentiation states, the predominant phenotype should be targeted to overcome tumor resistance. In the TME, the predominant T_reg_ cell phenotypes associated with tumor cells were immunosuppressive. These included TNFR and IL-10 phenotypes, which also expressed CD30; hence, targeting CD30^+^ T_reg_ cells could substantially overcome T_reg_ cell-mediated resistance to tumor growth.

Our data also highlight the interplay between PD-1 and STAT5 signaling. We have previously shown that PD-1 can inhibit STAT5 signaling in group 2 innate lymphoid cells, and this molecular mechanism is conserved in T_reg_ cells. We found that PD-1 could inhibit STAT5 phosphorylation in T_reg_ cells, albeit with a subtle effect, which then prevented expression of CD30 in these cells. There have been contradictory findings regarding the outcomes of PD-1 signaling in T_reg_ cells in general, with some studies reporting inhibition of CD28 signaling, whereas others have shown TCR blockade. However, none of the studies considered the IL-2 that was present within the culture system or produced by contaminating T cells in the culture or T_reg_ cells undergoing functional plasticity. By using an anti-IL-2 blocking antibody, we showed that in the absence of IL-2, CD30 is not induced in T_reg_ cells. Hence, IL-2 through STAT5 drives CD30 expression, which is inhibited by PD-1 but only in the presence of anti-IL-2. Hence, within the TME resistance landscape, combination therapies targeting checkpoint receptors that are driven by IL-2 signaling could substantially dampen T_reg_ cell function in the TME.

Our unbiased scRNA-seq analysis showed that CD30 expression post PD-1/PDL1 blockade was preserved in human T_reg_ cells. However, our work using human samples was limited by the sample number; further confirmatory phenotyping could enhance the validity of our findings regarding the use of CD30 as a combination drug in melanoma patients who develop resistance. Of note, in silico analyses of published datasets show that CD30 is a predictive biomarker of survival in human cancers.

In summary, using unbiased analysis and murine validation studies, we report on the mechanistic role of PD-1 in T_reg_ cell biology and function. This report consolidates previous contradictory findings and provides mechanistic insight into the importance of T_reg_ cells within the TME in anti-PD-1 resistance.

## Methods

### Mice

C57BL/6, CD45.1 C57BL/6 and *Rag1*^*−/−*^ mice were purchased from Charles River Laboratories, and *Foxp3*^*RFP*^ and *Foxp3*^*ERT2-Cre-eGFP*^ mice were purchased from Jackson Laboratory. C57BL/6 *Pdcd1*^*−/−*^ (*Pd1*^*−/−*^) mice were provided by L. Chen (Yale University School of Medicine) and were further bred to *Foxp3*^*RFP*^ mice to generate *Pd1*^*−/−*^*Foxp3*^*RFP*^ mice. *Pd1*^*fl/fl-DTR-Tdtomato*^ (*Pd1*^*fl/fl*^) mice were engineered by Ozgene. To ablate PD-1-expressing cells, *Pd1*^*fl/fl*^ mice were given up to ten doses of 10 μg kg^−1^ of diphtheria toxin from *Corynebacterium diphtheriae* (Sigma-Aldrich) in PBS via intraperitoneal (i.p.) injection. *Pd1*^*fl/fl*^ mice were further bred to *Foxp3*^*ERT2-Cre-GFP*^ (*Foxp3*^*ERT2cre*^) mice to generate *Pd1*^*fl/fl*^*Foxp3*^*ERT2cre*^ and *Pd1*^*fl/fl*^*Foxp3*^*ERT2cre+/−*^ mice. To induce Cre-mediated PD-1 excision, *Pd1*^*fl/fl*^*Foxp3*^*ERT2cre*^ mice were given up to five doses of 1 mg tamoxifen (Sigma-Aldrich) in corn oil (Sigma-Aldrich) via i.p. injection. All mice were bred and maintained in a pathogen-free facility at Newcastle University under a project license approved by the Home Office. Male and female mice aged 8 to 12 weeks were used for all mouse strains, except for *Foxp3*^*ERT2cre*^ and *Pd1*^*fl/fl*^*Foxp3*^*ERT2cre+/−*^ mice, where only females were used. All mouse experimental procedures were performed at Newcastle University incorporating the NC3R guidelines for animal research, and the data are presented according to the ARRIVE guidelines. Mice were maintained on a 12-h light–dark cycle, at a temperature of 21 ± 2 °C and humidity of 55 ± 5%. Mice were maintained in individually ventilated cages under positive pressure.

### Patient characteristics

Human PBMCs from healthy donors were derived from NHS Blood and Transplant in Newcastle upon Tyne. Patients with stage IV metastatic melanoma were recruited from Royal Victoria Infirmary in Newcastle upon Tyne under Research Ethics Committee reference 24/NE/0014. Patients were treated with a combination therapy of ipilimumab and nivolumab. Blood samples were collected at the subsequent posttreatment clinic visit, at which point the antibody plasma concentration was three half-lives for both antibodies. All samples were collected after informed consent had been provided. In keeping with the literature,^36^ the effects of anti-PD-1 treatment were deemed minimal in this cohort, and the samples were labeled as ‘baseline’ samples. We also mined a publicly available dataset that included TILs from treatment-naive patients^37^ and patients treated with anti-PD-1 therapy, comprising both baseline and posttreatment samples. In this cohort, both responders and nonresponders to anti-PD-1 monotherapy were used for analysis. For responders, only posttreatment biopsy samples were used, to ensure comparability with the nonresponders^37^.

### Reagents

Mouse complete medium was made up of DMEM (Gibco) supplemented with 10% fetal bovine serum (FBS), 2 mM l-glutamine (Corning), 0.1 mM nonessential amino acids, 1 mM sodium pyruvate (Sigma-Aldrich), 50 µM 2-mercaptoethanol, and penicillin and streptomycin (100 units ml^−1^ penicillin, 100 µg ml^−1^ streptomycin; Corning). Melanoma culture media was made up of DMEM (Gibco) supplemented with 10% FBS and penicillin and streptomycin (100 units ml^−1^ penicillin, 100 µg ml^−1^ streptomycin). Flow cytometry staining (FACS) buffer was made up of PBS supplemented with 0.5% bovine serum albumin (BSA) (Sigma-Aldrich) and 0.01% sodium azide. Miltenyi buffer was made up of PBS supplemented with 0.5% BSA and 2 mM EDTA (Sigma-Aldrich). Human PBMC culture medium was made up of X-VIVO 15 with gentamicin and phenol red (Lonza) formulated with 5% FBS. All murine and human antibodies were obtained from BioLegend unless otherwise stated.

### Tumor model

The B16 melanoma model was set up using established protocols^6,33^. Mice were inoculated with 3 × 10^5^ B16F10 melanoma cells via subcutaneous injection into the flank. Tumors were measured daily from day 5 postinoculation, and tumor volumes were calculated using the following equation: volume = π/6 × 0.5 × length × width^2^. In certain experiments, *Pd1*^*fl/fl*^ and *Pd1*^*fl/fl*^*Foxp3*^*ERT2cre*^ mice were used and treated with PBS or 1 mg tamoxifen in corn oil, respectively, via i.p. injection for 5 consecutive days before tumor reconstitution. For T_reg_ cell adoptive transfer experiments, *Rag1*^*−/−*^ mice were reconstituted with 5 × 10^4^ sorted CD4^+^CD25^*−*^ cells from CD45.1 WT mice as T_eff_ cells, along with 5 × 10^4^ sorted CD4^+^FoxP3^RFP^ cells as T_reg_ cells from either *Foxp3*^*RFP*^ or *Pd1*^*−/−*^*Foxp3*^*RFP*^ mice via intravenous injection at day 8 postinoculation. In certain experiments, mice were treated with 0.1 mg isotype control IgG2a (2A3, Bio X Cell) or anti-CD153 (RM153, 2BScientific) via i.p. injection five times at 2-day intervals, starting from day 9. Mice were euthanized when tumor volumes reached 800–1,500mm^3^.

### Flow cytometry

Single-cell suspensions from the indicated organs were stained with a Live/Dead fixable dead cell staining kit (Invitrogen) per the manufacturer’s instructions. Surface staining was performed for 30 min at 4 °C in FACS buffer with the following antibodies: CD3ε (clone: 145-2C11), CD30 (clone: mCD30.1), CD4 (clone: GK1.5), CD44 (clone: IM7), CD45.1 (clone: A20), CD45.2 (clone: 104), CD62L (clone: MEL-14), GARP (clone: YGIC86, eBioscience), GITR (clone: DTA-1, BD Biosciences), Nrp1 (clone: 3E12), PD-1 (clone: 29F.1A12), TIGIT (clone: 1G9), CD25 (clone: PC61), CD119 (clone: 2E2, eBioscience), ST2 (clone: DJ8, mdbioproducts), NK1.1 (clone: PK136), CD11c (clone: N418), PD-L1 (clone: 10F9G2), PD-L2 (clone: TY25), CD86 (clone: GL-1), CD80 (clone: 16-10A1), IA/IE (clone: M5/114.15.2), H2Kb (clone: AP6-88.5) and CD16/32 (clone: 93). For intracellular cytokine staining, cells were stimulated with a cell-stimulation cocktail containing protein transport inhibitors (eBioscience) for 4 h at 37 °C. Cells were then fixed and permeabilized using a FoxP3 Transcription Factor Staining Buffer Set (eBioscience) per the manufacturer’s instructions. Intracellular staining was performed overnight at 4 °C in 1× permeabilization buffer (eBioscience) with the following antibodies: Bcl2 (clone: 10C4, eBioscience), CD152 (clone: UC10-4B9), FoxP3 (clone: FJK-16S, eBioscience), Ki67 (clone: SolA15, eBioscience), Gata3 (clone: TWAJ, eBioscience), RORγt (clone: AFKJS-9, eBioscience), Tbet (clone: 4B10), Helios (clone: 22F6), IFNγ (clone: XMG1.2), IL-10 (clone: JES5-16ES), IL-17A (clone: TC11-18H10.1), GzmB (clone: QA16A02) and CD16/32 (clone: 93).

Human PBMCs were stained with a Live/Dead fixable dead cell staining kit (Invitrogen) per the manufacturer’s instructions. Cells were stained for 30 min at 4 °C with surface antibodies follows: CD4 (clone: RPA-T4), CD30 (clone: BY88), GARP (clone: 7B11), GITR (clone: 108-17) TIGIT (clone: A15153G) and CD16/32 (clone: 3G8, FUN-2 and 10.1). Cells were then fixed and permeabilized using a FoxP3 staining kit (eBioscience) per the manufacturer’s instructions. Intracellular staining was performed with antibodies including FoxP3 (clone: 206D) and CD152 (clone: BNI3) overnight at 4 °C.

Flow cytometry was performed using a BD LSR Fortessa X20. Sort purification was performed on a BD FACSAria Fusion. All data were analyzed using FlowJo 10 (Tree Star). Graphs and statistical analyses were done with SPICE 6.1 (https://niaid.github.io/spice/) and Prism v.10 (GraphPad Software).

### Annexin V/7-AAD staining

T_reg_ cells (CD4^+^CD25^+^) were isolated from *Foxp3*^*RFP*^ or *Pd1*^*−/−*^*Foxp3*^*RFP*^ mice using a CD4^+^CD25^+^ Regulatory T Cell Isolation Kit (Miltenyi Biotec) per the manufacturer’s instructions. Isolated T_reg_ cells were cultured in mouse complete medium at 1 × 10^6^ cells ml^−1^ and stimulated with 0.5 μg ml^−1^ anti-CD3 (clone: 145-2C11), 2 μg ml^−1^ anti-CD28 (clone: 37.51) and 80 ng ml^−1^ IL-2 (Miltenyi Biotec) for 72 h. After washing with cold PBS, T_reg_ cells were stained with 5 μl Annexin V and 5 μl 7-AAD in 100 μl annexin V binding buffer (BD Biosciences) for 15 min in the dark at room temperature and analyzed by flow cytometry within 1 h.

### Mouse and human PBMC cell preparation for BD Rhapsody

BD Rhapsody single-cell technology was utilized. For murine samples, isolated B16F10 melanoma TILs from C57BL/6 WT and *Pd1*^*−/−*^ mice were stained with lineage markers for 20 min at 4 °C as follows: CD3ε (clone: 145-2C11), CD4 (clone: GK1.5), CD5 (clone: 53-7.3), CD8α (clone: 53-6.7), CD11b (clone: M1/70), CD11c (clone: N418), CD19 (clone: 6D5), CD49b (clone: DX5), Ter119 (clone: TER-119), F4/80 (clone: BM8), B220 (clone: RA3-6B2) and Gr1 (clone: RB6-8C5). After washing with Miltenyi buffer, cells were incubated with anti-Thy1 (clone: 30-H12) and streptavidin-Percp cy5.5 for 20 min at 4 °C. Cells were washed again and incubated with AbSeq antibodies and unique sample tags using a BD Mouse Single-Cell Multiplexing Kit (BD Biosciences) for 60 min on ice. The following AbSeq antibody-oligos were used: CD25, CD103, CD119, CD37, CD223, CD272, CD273, CD274, CD278, CD279, IL-17Rb, IL-23R, IL-33R, CD335, CD3 and NK1.1. After washing three times, cells were stained with DAPI and flow-enriched for the Lineage^+^ Thy1^+^ population consisting of T cells, innate immune cells and NK cells. For human samples, PBMC processing and Rhapsody experiments were performed as previously described^38,39^.

### Single-cell library preparation and sequencing

Single-cell libraries were prepared according to the protocol provided by BD Biosciences. Briefly, a pooled sample tag, AbSeq and mRNA library was sequenced with minimum read lengths of 51 bp (R1) and 71 bp (R2) and a 200-cycle sequencing protocol. Libraries were sequenced on a NovaSeq 6000 (Illumina) using an S2 Reagent Kit v.1.5 (200 cycles).

### Preprocessing of scRNA-seq data and dimensionality reduction

For murine TIL and human PBMC scRNA-seq datasets, FastQ files were processed using the BD Rhapsody analysis pipeline (https://www.sevenbridges.com/bdgenomics/), which generated unique molecular identifier (UMI) counts. In RStudio (R v.3.3), the Seurat package (v.5.0.3) was used to further analyze transcriptomic and protein expression data. For murine samples, cells were filtered to include those with ≥10 UMIs and 20–300 detected genes per cell. For human samples, cells were filtered to include those with 1,200–60,000 UMIs and 50–200 detected genes per cell. Multiplets and indeterminates were removed from downstream analysis. Expression matrices were log-normalized, and the top 2,000 variable features were identified. Samples were integrated using an anchor set and scaled by *z*-score conversion. Dimensionality reduction (npcs = 50) was performed using principal component analysis (PCA). The top ten and top 20 principal components (PCs) were used to compute UMAP for murine and human scRNA-seq. A *k*-nearest neighbor graph (KNN) was constructed using the FindNeighbors function (k.param = 20, dims = 1:10 for murine and 1:20 for human, annoy.metric = ‘euclidean’), and Louvain clustering (resolution = 0.6) was performed.

For mining of publicly available datasets, the data files were preprocessed^37,40^, and the processed gene expression data were retrieved from the National Center for Biotechnology Information (NCBI) Gene Expression Omnibus (GEO) database (http://www.ncbi.nlm.nih.gov/geo). In RStudio, the Seurat package was used to identify the top 2,000 variable features. Dimensionality reduction (npcs = 50) was performed using PCA. The top ten and top 20 PCs were used to compute UMAPs for treatment-naive melanoma patients and for anti-PD-1-treated melanoma patients at baseline and after treatment, respectively. A KNN graph was constructed using the FindNeighbors function (k.param = 20, dims = 1:10 for treatment-naive melanoma patients and 1:20 for anti-PD-1-treated melanoma patients, annoy.metric = ‘euclidean’). Louvain clustering was performed with a resolution of 0.2 for treatment-naive melanoma patients and 0.1 for anti-PD-1-treated melanoma patients.

### scRNA-seq analysis

For scRNA-seq analysis of murine TILs, cluster identities were assigned using the ScType function based on cell-type-specific gene sets (Supplementary Table 3a), resulting in nine clusters (basophils, T_eff_ cells, macrophages, megakaryocytes, myeloid DCs, B cells, NK cells, plasmacytoid DCs and T_reg_ cells) in murine TILs. The T_eff_ cell cluster was further subclustered into CD4 central memory T, CD4 T_eff_, CD8 central memory T and CD8 T_eff_ cell clusters. For scRNA-seq analysis, of human PBMCs, cluster identities were assigned using the ScType function based on cell-type-specific gene sets (Supplementary Table 6a). In addition, cells were annotated using a manual review process based on algorithmically defined marker gene expression for each cluster with the FindAllMarkers function (method = ‘Wilcoxon’ with Bonferroni correction), together with protein expression data, resulting in 16 clusters of human PBMCs (B cells, CD8^+^ Tc1 cells, CD11c^+^CD14^+^, CD11c^+^CD16^+^, CD4^+^ T_eff_, CD8^+^ T_eff_, γδ, CD4^+^ memory T, CD8^+^ memory T, CD4^+^ T naive, CD8^+^ T naive, CD161^+^ NK, NK, CD8^+^ Tc17, T_reg_ and other). For public datasets, cluster identities were annotated based on algorithmically defined marker gene expression for each cluster with the FindAllMarkers function (method = ‘Wilcoxon’ with Bonferroni correction). This process revealed nine clusters (CD4 T cells, CD8 T cells, CD4^−^CD8^−^ T cells, B cells, macrophages, endothelial cells, cancer-associated fibroblasts, melanoma cells and NK cells) for treatment-naive patients and six clusters (CD4 T cells, CD8 T cells, B cells, monocytes and macrophages, DCs, and NK and NK T cells) for anti-PD-1-treated patients in which the top preferentially expressed genes (adjusted *P* < 0.001) included multiple known markers of particular cell types. The CD4 T cell cluster was further divided into CD4 T cells without FoxP3 expression and T_reg_ cells with FoxP3 expression in both datasets. Gene expression of clusters was visualized as a dot plot using the JJDotPlot function in the scRNAtoolVis package (v.0.1.0).

### Differential gene expression and pseudotime trajectory analysis

Differential gene expression was determined using the FindMarkers function (method = ‘Wilcoxon’ with Bonferroni correction). For this analysis, AbSeq antibodies were excluded. Genes were considered to be differentially expressed if they achieved log_2_ (fold change) > 0.585 and adjusted *P* < 0.05. The results were illustrated as a volcano plot using the ggplot2 (v.3.5.0) package. To overcome the limitations of sample pooling, mRNA expression levels of coinhibitory receptors were obtained and plotted to show expression in each individual sample. The average mRNA expression of selected genes in T_reg_ cells across individuals was calculated using the mean function with summarise from the dplyr package (v.1.1.4). Unpaired Student’s *t*-test was performed to measure differences between groups. In addition, single-cell mRNA expression was visualized using violin plots generated with the VlnPlot function in the Seurat package, and clustered Wilcoxon rank-sum test^41^ was performed using the clusWilcox.test function (method = ‘ds’) in the clusrank (v.1.0-4) package to overcome sample pooling effects while measuring differences in coinhibitory receptor expression between two groups. GO overrepresentation analysis and GSEA were performed using the clusterProfiler (v.4.10.1) package. Gene symbols were converted into Entrez ID using the biomaRt package (v.3.18). The enriched GO terms were acquired from the list of differentially expressed genes using the enrichGO function and visualized as a dot plot using the dotplot function. Statistical analysis was performed using Fisher’s exact test with false discovery rate (FDR) correction, and adjusted *P* values are shown. GSEA was conducted using the gseGO function; genes were ranked using the default Kolmogorov–Smirnov test with FDR correction, and a *P* value cutoff of 0.05 was used to identify significant GO terms. Pseudotime single-cell trajectory analysis was performed using the Monocle2 plugin in SeqGeq.

### Cell–cell communications

To determine cell–cell interactions and signaling patterns, we applied the cell communications CellChat (v.2) R package to our single-cell analysis (https://github.com/sqjin/CellChat)^42^. CellChat cross-references a ligand–receptor interaction database (CellChatDB) including extracellular matrix–receptor interactions, cell–cell contact interactions and secreted signals. To identify significant cell–cell interactions, differentially expressed ligands and receptors were first determined using the identifyOverExpressedGenes and identifyOverExpressedInteractions functions with default parameters. Communications with fewer than three cells were excluded with the filterCommunication function. The communication probability was calculated with the computeCommunProbPathway function, and significant communications were identified using a permutation test. An aggregated cell–cell communication network was then compiled using the aggregateNet function; this network was visualized as a circle plot using the netVisual_circle function and as a chord plot using the netVisual_chord_gene function. Network centrality scores were then calculated using the netAnalysis_computeCentrality function, and the role of each cell type as a signal sender or receiver within the inferred communication network were quantified using the netAnalysis_signalingRole_heatmap function. Cophenetic and Silhouette values were evaluated by using the selectK function to identify the optimal number of communication patterns of cell types. Outgoing and incoming patterns were then determined using the identifyCommunicationPatterns function to reveal how the sender cells coordinated with certain outgoing signaling pathways to drive communication or how the target cells responded to incoming signaling pathways.

### PD-L1 molecular mechanistic studies

Cell-culture 96-well plates were prepared by coating with either 5 μg ml^−1^ anti-CD3 alone or 5 μg ml^−1^ anti-CD3 and recombinant PD-L1 Fc chimera protein for 3 h at 37 °C. The wells were washed with PBS three times. T_reg_ cells were then seeded in the wells and cultured in mouse complete medium at 1 × 10^6^ cells ml^−1^, along with 2 μg ml^−1^ anti-CD28 (clone: 37.51). In certain wells, 10 μg ml^−1^ anti-PD-1 (clone: RMP1.14) was added to block PD-1–PD-L1 interaction. After 72 h, cells were washed with complete medium and further stimulated with either 10 μg ml^−1^ anti-IL-2 (clone: S4B6, Invitrogen) alone or 10 μg ml^−1^ anti-IL-2 and 80 ng ml^−1^ IL-2 (Miltenyi Biotec) for 24 h in a humidified incubator at 37 °C with 5% CO_2_. Cells were then harvested for flow cytometry and quantitative reverse transcription polymerase chain reaction (RT–qPCR) assays.

### T_reg_ cell suppressor assays

The T_reg_ cell suppressor assay was performed as previously published^27^. CD90.2T cells were purified from splenocytes isolated from *Foxp3*^*RFP*^ or *Pd1*^*−/−*^*Foxp3*^*RFP*^ mice using CD90.2 MicroBeads (Miltenyi Biotec) per the manufacturer’s instructions. Cells were stained with anti-CD4, and cell viability was assessed by DAPI before sorting. Sorted T_eff_ cells were stained in PBS with cell trace violet. Then, 5 × 10^4^ T_eff_ cells and the indicated ratios of sorted T_reg_ cells from either *Foxp3*^*RFP*^ or *Pd1*^*−/−*^*Foxp3*^*RFP*^ mice were stimulated using 0.5 μg ml^−1^ anti-CD3 along with irradiated splenocytes from C57BL/6 WT mice as antigen-presenting cells using a 4:1 ratio of antigen-presenting cells to T_eff_ cells. Cells were cultured in complete medium for 72 h at 37 °C with 5% CO_2_, and proliferation was assessed as the dilution of cell trace violet using flow cytometry.

### Phospho-STAT5 assays

Phospho-STAT5 assays using coated PD-L1 Fc chimera proteins were performed using our established protocols^4,18^. Cell-culture plates were prepared by coating with 5 μg ml^−1^ anti-CD3 and 5 μg ml^−1^ recombinant PD-L1 Fc chimera protein for 3 h at 37 °C. Control wells were coated with anti-CD3 alone. The wells were washed with PBS three times. Isolated T_reg_ cells were then cultured in mouse complete medium at 1 × 10^6^ cells ml^−1^ and incubated with 2 μg ml^−1^ anti-CD28 and 10 μg ml^−1^ anti-IL-2 for 48 h in a humidified incubator at 37 °C with 5% CO_2_. In certain wells, 10 μg ml^−1^ anti-PD-1 was added to block PD-1–PD-L1 interaction. After 48 h, cells were washed with complete medium and further stimulated with 80 ng ml^−1^ IL-2 for 15 min at 37 °C. Cell fixation and permeabilization were performed using BD Phosflow Lyse/Fix buffer and BD Phosflow Perm Buffer III per the manufacturer’s instructions. Cells were then stained with anti-pSTAT5 (clone: 47; BD Biosciences) for 20 min at room temperature and analyzed by flow cytometry

### RNA extraction from T_reg_ cells

mRNA was extracted from T_reg_ cells using mRNeasy Kits (Qiagen) per the manufacturer’s protocol. cDNA synthesis from mRNA was carried out using TaqMan Reverse Transcription Reagents (Applied Biosystems).

### RT–qPCR

cDNAs were used as the template for RT–qPCR using a LightCycler 480 II (Roche). TaqMan Gene Expression assays (FAM) were purchased from Life Technologies Thermo Fisher Scientific for quantification of mouse CD30 mRNA (Mm00437140_m1 (*Tnfrsf8*)), as well as actin mRNA (Mm00607939_s1 (*Actb*)), which was used as a housekeeping control. All TaqMan assay RT–qPCRs were performed using TaqMan Fast Advanced Master Mix (Applied Biosystems). The RT–qPCR reaction included an initial step of 10 min at 95 °C followed by 50 cycles of 15 s denaturation at 95 °C and then 60 s annealing and extension at 60 °C.

### RT–qPCR data analysis

Results were processed using LightCycler 480 software. Samples were assigned to control (WT T_reg_) and experimental (*Pd1*^*−/−*^ T_reg_) groups. Cycle threshold (*C*_t_) values were then normalized, with *Actb* as a reference gene, using the following formula: Δ*C*_t_ = average *C*_t_ (*Tnfrsf8*) *−* average *C*_t_ (*Actb*). The difference between the Δ*C*_t_ values for the WT T_reg_ cells and *Pd1*^*−/−*^ T_reg_ cells was calculated using the formula ΔΔ*C*_t_ = Δ*C*_t_ (*Pd1*^*−/−*^ T_reg_) *−* Δ*C*_t_ (WT T_reg_). The expression fold change was then calculated as follows: fold change = $2^{-\Delta\Delta C_{\rm{t}}}$.

### CosMx tissue processing

Tumors were harvested from B16F10 melanoma-bearing *Rag1*^*−/−*^ mice reconstituted with CD45.1^+^ WT T_eff_ cells and either WT T_reg_ cells or *Pd1*^*−/−*^ T_reg_ cells and fixed in 10% neutral buffered formalin (Sigma-Aldrich) overnight. Tumor tissues were embedded in paraffin, punched as 3-mm biopsies and mounted onto the centers of Leica Bond Plus slides. Formalin-fixed paraffin-embedded slides were air-dried at room temperature after sectioning. Hematoxylin and eosin staining of slides was performed by NovoPath at Newcastle Hospitals, and the stained slides were observed under a Zeiss Axio Imager 2 and analyzed using ZEISS ZEN software to assess tissue block quality and select FOVs.

### CosMx image acquisition and cell segmentation

CosMx experiments were performed by NanoString as part of the Technology Access Program. Briefly, standard methods for fluorescence in situ hybridization were applied by NanoString to formalin-fixed paraffin-embedded tissue slides to expose RNA targets as previously described^43^. Fluorescent bead-based fiducials fixed to the tissue were introduced to provide an optical reference for cyclic image acquisition, followed by hybridization of RNA-specific probes and antibodies. After washing, each sample was assembled into a flow cell and loaded onto a spatial molecular imaging (SMI) instrument for RNA readout and morphological imaging. Raw images were transformed to decoded RNA transcripts at subcellular resolution through a workflow including (1) three-dimensional primary image processing to identify and register reporter spots; (2) decoding of reporter spots to RNA transcripts with registered *x*, *y* and *z* spatial locations; (3) outlining of nucleus and cell boundaries with DAPI and antibodies after cyclic reporter readout for morphology-based cell segmentation; and (4) assigning RNA transcripts to single cells.

### CosMx data analysis

CosMx SMI data and flat files were exported from AtoMx SIP to a QWS S3 Bucket. The CosMx SMI data output consisted of a Seurat object, TileDB array, and a ‘Flow Cell ID’ folder containing raw decoded files and images for each FOV, whereas the flat file output consisted of a count matrix, cell metadata, global transcripts, global cell boundaries and global FOV positions. Both output files were downloaded using the S3 console and used for data analysis. Samples were loaded into RStudio (R v.4.3.3) using the LoadNanostring function, and expression profiles of cells were clustered using the Seurat package (v.5.0.0). Briefly, data were normalized and scaled using the SCTransform function. Dimensionality reduction (number of principle components, npcs = 5) was performed using PCA, and the top five PCs were used to compute UMAPs. A KNN graph was constructed using the FindNeighbors function (k.param = 20, dims = 1:5, annoy.metric = ‘euclidean’), followed by Louvain clustering with a resolution of 0.2. Cluster identities were assigned using the ScType function based on cell-type-specific gene sets and published papers on cell identification in skin and tumor tissue^44,45^, resulting in five clusters in the murine TME (basal cells, immune cells, Schwann cells, tumor cells and melanocytes). The ‘immune cells’ cluster was further subclustered into monocytes, DCs, innate lymphoid cells, mast cells, T_reg_ cells, NK cells, macrophages and CD4^+^ T cells. Each FOV region was zoomed in and visualized using the Crop function. Using ImageDimPlot function, we overlaid the cell identities onto the FOV images. Spatial expression pattern of individual molecules was visualized using the ImageFeaturePlot function. Visualization of gene expression, calculation of average gene expression, differential gene expression analysis and enrichment analysis were performed as described for the scRNA-seq analysis. Clustered Wilcoxon rank-sum tests were performed using the clusWilcox.test or pairwise.clusWilcox.test function (method = ‘ds’) in clusrank (v.1.0-4) to measure differences in coinhibitory receptor expression between two groups or among multiple groups, with FDR correction applied for comparisons involving more than two groups.

### CosMx spatially resolved cell–cell interaction analysis

The T_reg_ cell population was further divided into several groups based on spatial location and cell interactions in FOVs using specific Cell_IDs. The numbers of physical interactions of T_reg_ cells with other cell types in the FOV images were manually counted. Using the FindAllMarkers function (method = ‘Wilcoxon’ with Bonferroni correction), specific genes in each T_reg_ cell subset interacting with neighboring cells or alone within the FOV were determined by visually annotating cells. T_reg_ cell-specific genes were further subdivided into either SR or LR communication categories based on their biological function by manual annotation. Genes involved in cell–cell contact were annotated as SR, and secretion or signaling genes were annotated as LR. The SR category consisted of genes for cell surface receptors, proliferation markers and apoptotic markers, whereas LR genes encoded cytokines, chemokines, transcription factors that could drive cytokine and chemokine production, and so on.

### CHIP–seq analysis

The STAT5 CHIP–seq dataset was derived from NCBI GEO under accession number GSE207265 (ref. ^29^). In brief, naive WT CD4^+^ cells were stimulated with 10 μg ml^−1^ plate-bound anti-CD3 (17A2, Bio X Cell) and anti-CD28 (37.51, Bio X Cell) in the presence of anti-mouse IL-2 (S4B6) for 48 h. Cells were then washed and cultured with 100 U ml^−1^ human IL-2 (NIH/National Cancer Institute Biological Resources Branch Preclinical Repository) for 48 h. Genome browser files were rendered with Integrated Genome Viewer.

### RNA-seq analysis

The RNA-seq dataset was derived from NCBI GEO under accession number GSE207265 (ref. ^29^). In brief, naive WT and *Stat5*^*−/−*^ CD4^+^ cells were cultured with 10 μg ml^−1^ plate-bound anti-CD3 (clone: 17A2) and anti-CD28 (clone: 37.51) in the presence of anti-mouse IL-2 (clone: S4B6) for 72 h. Cells were then washed and restimulated with an anti-TCR agonist with either anti-IL-2 or 100 U ml^−1^ human IL-2 (NIH/National Cancer Institute Biological Resources Branch Preclinical Repository) for 4 days. For GSEA-based ranking of TNFRSF family members, transcripts of the TNF family including *Tnfrsf8*, *Tnfrsf4*, *Tnfrsf13b*, *Tnfrsf11b*, *Tnfrsf18*, *Tnfrsf21*, *Tnfrsf1b*, *Tnfrsf9*, *Tnfrsf17*, *Tnfrsf19*, *Ltbr*, *Eda2r*, *Tnfrsf11a*, *Ngfr*, *Tnfrsf1a*, *Tnfrsf12a*, *Tnfrsf10a*, *Cd40*, *Tnfrsf13c*, *Fas*, *Tnfrsf25*, *Cd27* and *Tnfrsf14* were ranked by rank metric score.

### In vitro culture of melanoma patient PBMCs

Human PBMCs derived from patients with stage IV metastatic melanoma were plated at 1 × 10^6^ cells ml^−1^ in a 96-well U-bottomed plate. Cells were stimulated with 0.5 μg ml^−1^ anti-CD3 (clone: OKT3) and 20 μg ml^−1^ of the isotype control IgG2b (clone: MPC-11) or anti-PD-L1 (clone: 29E.2A3) for 72 h in a humidified incubator at 37 °C with 5% CO_2_.

### CD30 biomarker analysis

Analysis of CD30 as a biomarker in different cancers was performed on the R2 Genomics Analysis and Visualization Platform. The dataset analyzed was derived from The Cancer Genome Atlas and NCBI GEO under accession numbers GSE42568 (ref. ^46^) and GSE33114 (ref. ^47^). Kaplan–Meier survival curves with the default log-rank test were used to determine the optimum expression cutoff for survival analyses.

### Statistical analysis

All data shown are expressed as individual data points with the line at the mean unless otherwise stated. Error bars on graphs show the standard error of the mean. Paired or unpaired two-tailed Student’s *t*-test or two-tailed clustered Wilcoxon rank-sum test was used for comparisons between two groups; and one- or two-way analysis of variance corrected by Sidak’s multiple comparison test, Wilcoxon rank-sum test corrected by Bonferroni correction or clustered Wilcoxon rank-sum test corrected by FDR was used for comparisons among multiple groups.

### Reporting summary

Further information on research design is available in the Nature Portfolio Reporting Summary linked to this article.

## Online content

Any methods, additional references, Nature Portfolio reporting summaries, source data, extended data, supplementary information, acknowledgements, peer review information; details of author contributions and competing interests; and statements of data and code availability are available at 10.1038/s41590-025-02172-0.

## Supplementary information

Supplementary Information Supplementary Figs. 1–9.

Reporting Summary

Supplementary Tables 1–6 This file contains multiple tabs of supplementary tables, each clearly labeled in the spreadsheet.

## Data Availability

scRNA-seq data from treatment-naive and anti-PD-1-treated melanoma patients used in this paper were accessed via GEO under accession codes GSE72056 and GSE120575 for the Tirosh and Sade-Feldman datasets, respectively. All raw data from the mouse single-cell BD Rhapsody RNA-seq experiments and human single-cell BD Rhapsody experiments, along with the NanoString CosMx data, have been uploaded to GEO: scRNA-seq data have been deposited under accession code GSE273532, human scRNA-seq data under accession code GSE280319, and the NanoString CosMx SMI dataset (both the Seurat object and flat files) under accession code GSE273530. The flat files were used for the analysis presented in this manuscript. The CellChat database for mouse datasets used in the cell–cell communication analysis is available via GitHub at https://github.com/sqjin/CellChat.

## Code availability

The code used in analyzing the dataset is available via GitHub at https://github.com/JXLIM99/scRNAseq-and-Nanostring-Cosmx-SMI.
