# Supplementary material for: PD-1 receptor deficiency enhances CD30+ Treg cell function in melanoma
Source: Nat Immunol. 2025 Jun 2;26(7):1074–86. doi: 10.1038/s41590-025-02172-0 (PMC12208868; doi:10.1038/s41590-025-02172-0)
Supplement: Supplementary file 1 — Supplementary Figs. 1–9. [file 41590_2025_2172_MOESM1_ESM.pdf]

nature immunology

Supplementary information https://doi.org/10.1038/s41590-025-02172-0

PD-1 receptor deficiency enhances CD30^{+} T_{reg} cell function in melanoma

In the format provided by the authors and unedited

Supplementary Figures and Figure Legends

Supplementary Figure 1. Flow cytometry analysis of Foxp3^{+} and Foxp3^{-} cells in WT and *Pd1*$^{-/-}$ cohort in steady state, Related to Figure 1.

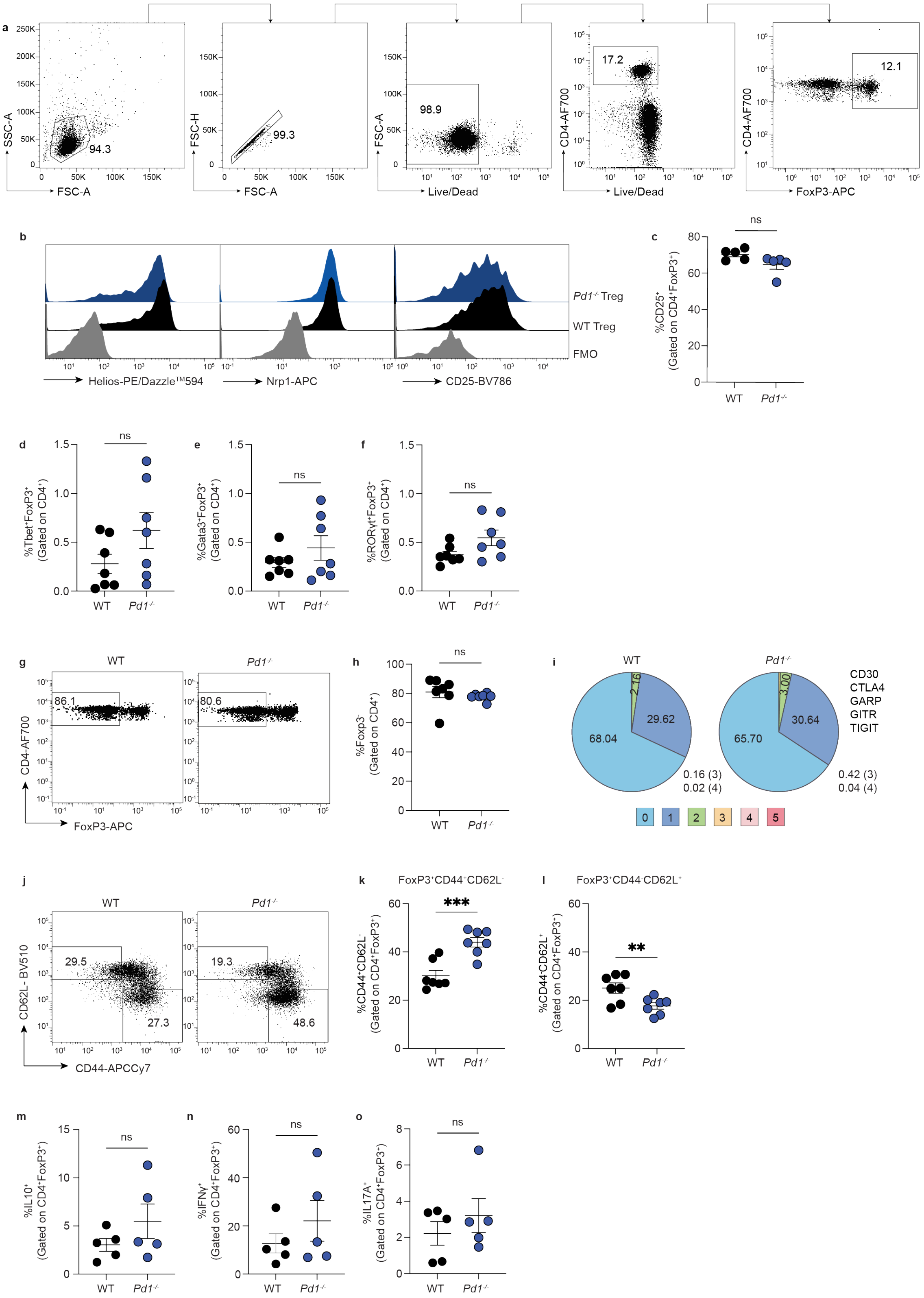

Immunophenotyping of $CD4^+$ T cells from healthy $Foxp3^{RFP}$ (WT) and $Pd1^{-/-}Foxp3^{RFP}$ ($Pd1^{-/-}$) mice. **A,** Representative flow cytometry plots showing gating strategy used to identify $CD4^+FoxP3^+$ Treg cells. **B,** Representative flow cytometry histograms showing Helios, Nrp1 and CD25 expression in Treg cells. **C,** Summary data showing frequency of CD25 (n=5 per group). **D-F,** Summary data showing frequency of $Tbet^+FoxP3^+$ cells, $GATA3^+FoxP3^+$ cells and $ROR\gamma t^+FoxP3^+$ cells (n=7 per group). **G**, Representative flow plot showing $CD4^+FoxP3^-$ T cells following gating strategy shown in A. **H**, Summary of the frequency of $CD4^+FoxP3^-$ T cells (n=7 per group). **I**, Boolean analysis of $CD4^+FoxP3^-$ T cells. **J**, Representative flow plots of CD44 and CD62L expression in Treg cells. **K**, Summary of the frequency of $CD4^+FoxP3^+CD44^+$ and **L**, $CD4^+FoxP3^+CD62L^+$ Treg cells (n=7 per group). **M-O**, Summary of IL-10, IFNγ and IL-17A expression in Treg cells (n=5 per group). Each data point represents an animal from independent experiment. Data shown are mean±SEM, a two-tailed unpaired Student's *t* test was performed. $^{**}P \leq 0.01$, $^{***}P \leq 0.001$; ns, not significant.

Supplementary Figure 2. Flow cytometry analysis of Foxp3⁺ cells in *Pd1^fl/fl^Foxp3^ERT2cre^* mice in steady state, Related to Figure 1

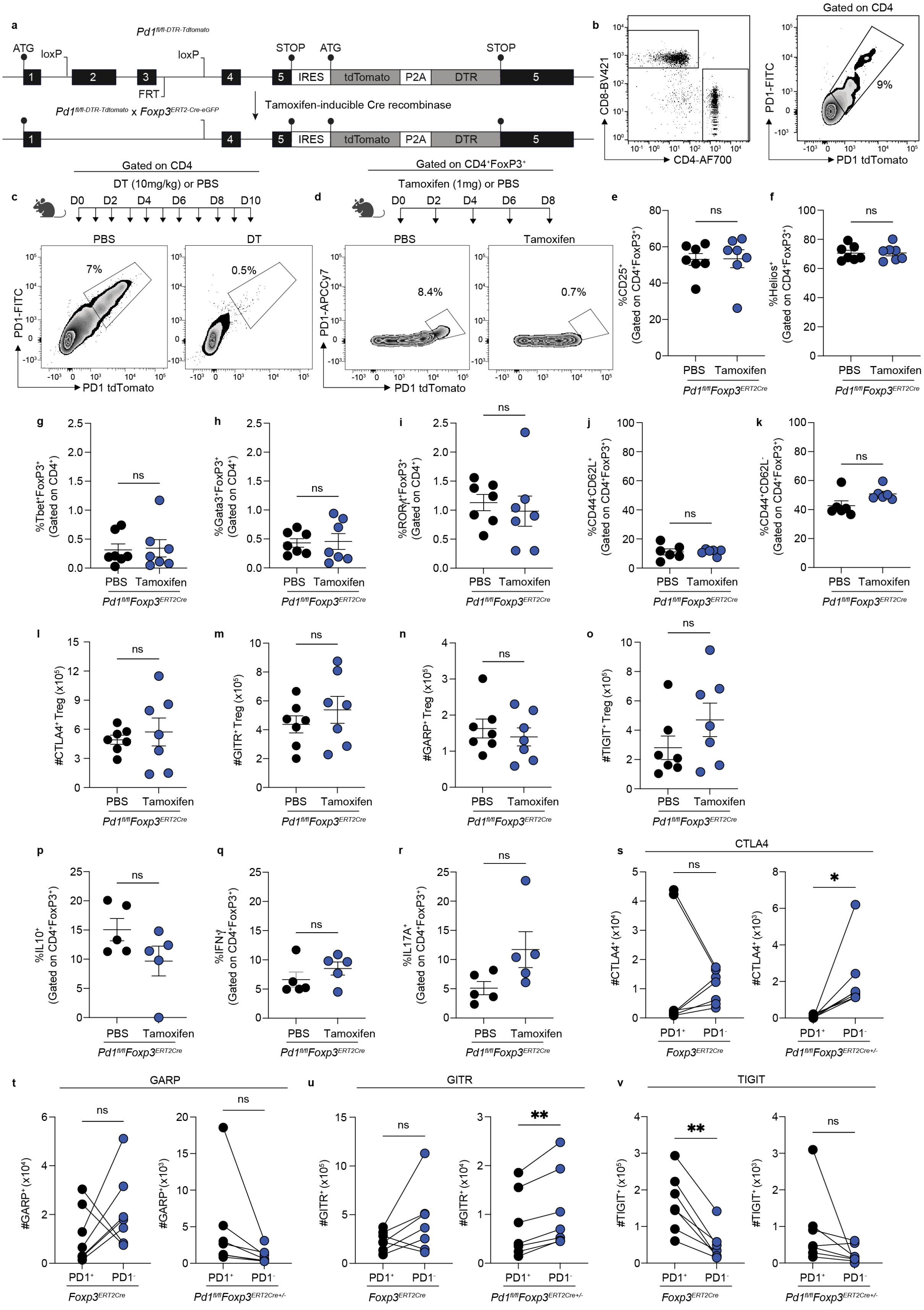

Pd1$^{fl/fl}$ animals were generated by Ozgene. **A,** *Pdcd1* gene locus in Foxp3-expressing cells when *Pd1*$^{fl/fl}$*Foxp3*^{ERT2Cre} mice treated with tamoxifen. **B,** Analysis of healthy *Pd1*$^{fl/fl}$ murine splenocytes. Representative flow cytometric plot of $CD4^+$ versus $CD8^+$ T cells (**left panel**) and PD1 antibody versus tdTomato gated on $CD4^+$ T cells (**right panel**). **C,** *Pd1*$^{fl/fl}$ mice were administered with PBS or diphtheria toxin (DT) for up to 10 dosages via IP. Representative flow cytometric plots of PD1 antibody versus tdTomato gated on $CD4^+$ cells treated with PBS **(left panel)** or treated with DT (**right panel**). **D,** *Pd1*$^{fl/fl}$*Foxp3*^{ERT2Cre} mice were administered with PBS or tamoxifen up to 5 dosages. Flow cytometric plots of PD1 antibody versus tdTomato expression in Treg cells in PBS (**left panel**) or tamoxifen-treated mice (**right panel**). **E-K**, Immunophenotyping of PBS and tamoxifen-treated *Pd1*$^{fl/fl}$*Foxp3*^{ERT2Cre} Treg splenocytes. **E-F,** Summary of CD25 and Helios expression in Treg cells (n=7 per group). **G-I**, Frequency of $Tbet^+FoxP3^+$ cells, $GATA3^+FoxP3^+$ cells and $ROR\gamma t^+FoxP3^+$ cells (n=7 per group). **J,** Summary of $CD4^+FoxP3^+CD62L^+$ cells and **K**, $CD4^+FoxP3^+CD44^+$ cells (n=6 per group). Absolute counts of **L**, $CTLA4^+$ Treg cells, **M**, $GITR^+$ Treg cells, **N**, $GARP^+$ Treg cells and **O**, $TIGIT^+$ Treg cells. **P-R**, Summary of IL-10, IFN-γ and IL-17A in Treg cells (n=5 per group). **S-V,** *Foxp3*^{ERT2Cre} and *Pd1*$^{fl/fl}$*Foxp3*^{ERT2Crehet} mice were administered with tamoxifen up to 5 dosages (n=7 per group). Absolute counts of **S**, $CTLA4^+$ Treg cells, **T**, $GARP^+$ Treg cells, **U**, $GITR^+$ Treg cells, **V**, $TIGIT^+$ Treg cells. Data shown are mean±SEM, each data point represents an independent experiment in **E-R** and each pair of data point represents an individual animal in **S-V**. A two-tailed unpaired Student's *t* test was performed in **E-R** and a two-tailed paired Student's *t* test in **S-V**. *$P \leq 0.05$, **$P \leq 0.01$; ns, not significant. Schematic illustrations were created in BioRender. Smith, K. (2025) https://BioRender.com/5f58wy2.

Supplementary Figure 3. *Pd1$^{-/-}$* Treg cells do not differ in proliferation or apoptosis phenotype in steady state.

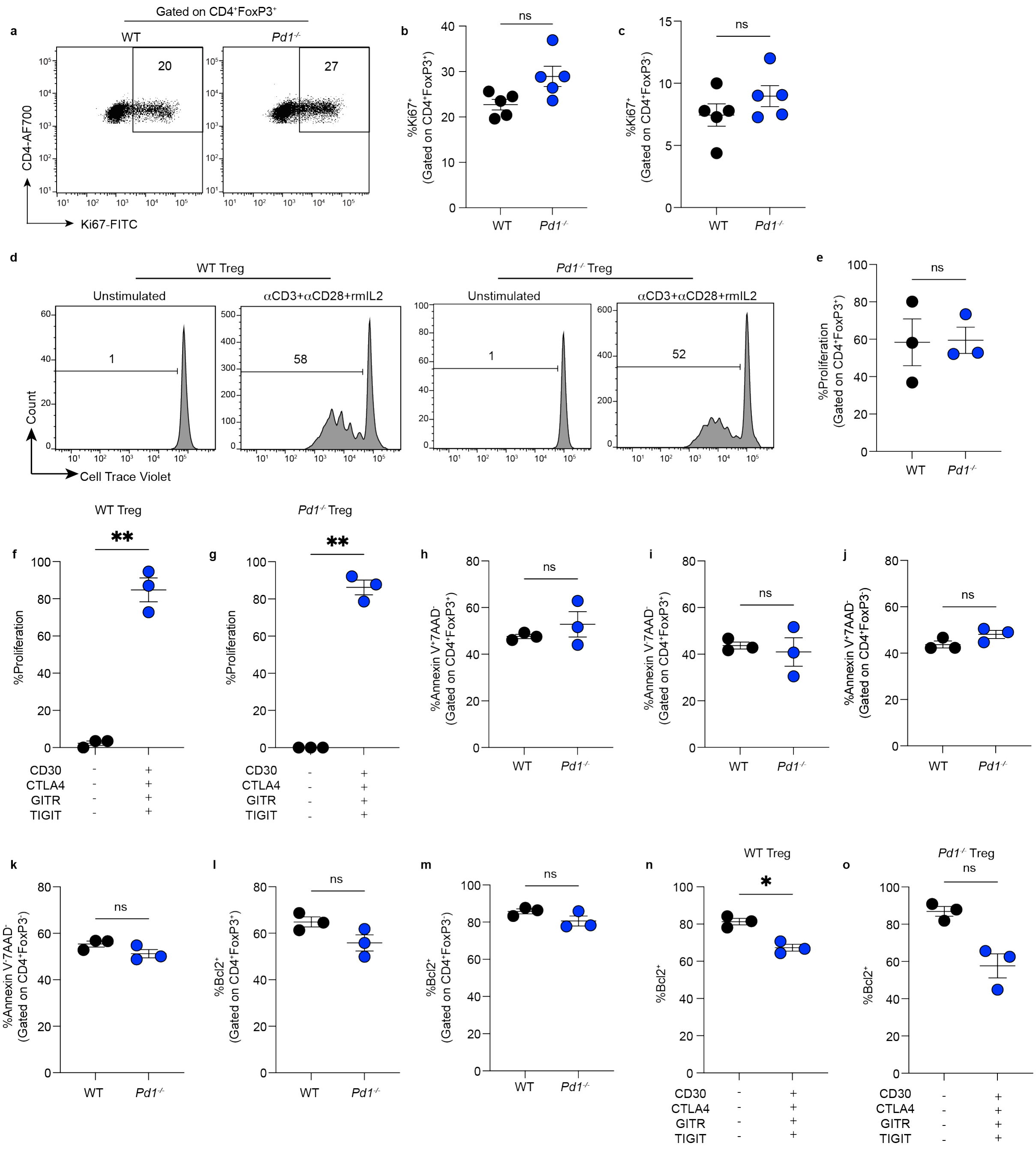

A-G, Analysis of proliferative signature of Treg cells and T effectors in spleens of healthy C57BL/6 (WT) and *Pd1$^{-/-}$* mice. **A**, Representative flow cytometry plot of Ki67 expression in gated $CD4^+FoxP3^+$ T cells. **B**, Summary data of the frequency of $Ki67^+$ cells in $CD4^+FoxP3^+$ T cell and **C**, $CD4^+FoxP3^-$ T cells (n=5 per group). **D,** Representative flow cytometry plots showing cell trace violet dilution at 72 h post *in-vitro* stimulation of isolated WT and *Pd1$^{-/-}$* Treg cells with αCD3/28 and rmIL-2. **E**, Summary data showing proliferation of $CD4^+FoxP3^+$ Treg cells (n=3 per group). **F-G,** Summary data showing proliferation of

$CD30^{+}CTLA4^{+}GITR^{+}TIGIT^{+}$ versus $CD30^{-}CTLA4^{-}GITR^{-}TIGIT^{-}$ Treg cells (n=3 per group). **H-K,** Analysis of anti-apoptotic phenotype of Treg cells and T effectors in spleens of healthy C57BL/6 (WT) and *Pd1*$^{-/-}$ mice (n=3 per group). **H-I**, Frequency of Annexin $V^{+}7AAD^{-}$ cells and Annexin $V^{-}7AAD^{-}$ cells in $CD4^{+}FoxP3^{+}$ T cells. **J-K**, Frequency of Annexin $V^{+}7AAD^{-}$ cells and Annexin $V^{-}7AAD^{-}$ cells in $CD4^{+}FoxP3^{-}$ T cells. **L-M**, Frequency of Bcl2 expression in $CD4^{+}FoxP3^{+}$ T cells and $CD4^{+}FoxP3^{-}$ T cells (n=3 per group). **N-O**, Frequency of Bcl2 expression in $CD30^{+}CTLA4^{+}GITR^{+}TIGIT^{+}$ versus $CD30^{-}CTLA4^{-}GITR^{-}TIGIT^{-}$ Treg cells (n=3 per group). Data shown are mean±SEM, each data point represents an animal from independent experiment in **B-C, E, H-J, K-M,** and data point within each group is matched to the same individual mouse in **F-G, N-O**. Statistical analyses were performed using a two-tailed unpaired Student's *t* test in **B-C, E, H-J, K-M** and a two-tailed paired Student's *t* test in **F-G, N-O**. *P ≤ 0.05, **P ≤ 0.01; ns, not significant.

Supplementary Figure 4. BD Rhapsody scRNA-seq analysis of Treg cells and T effectors, Related to Figure 2.

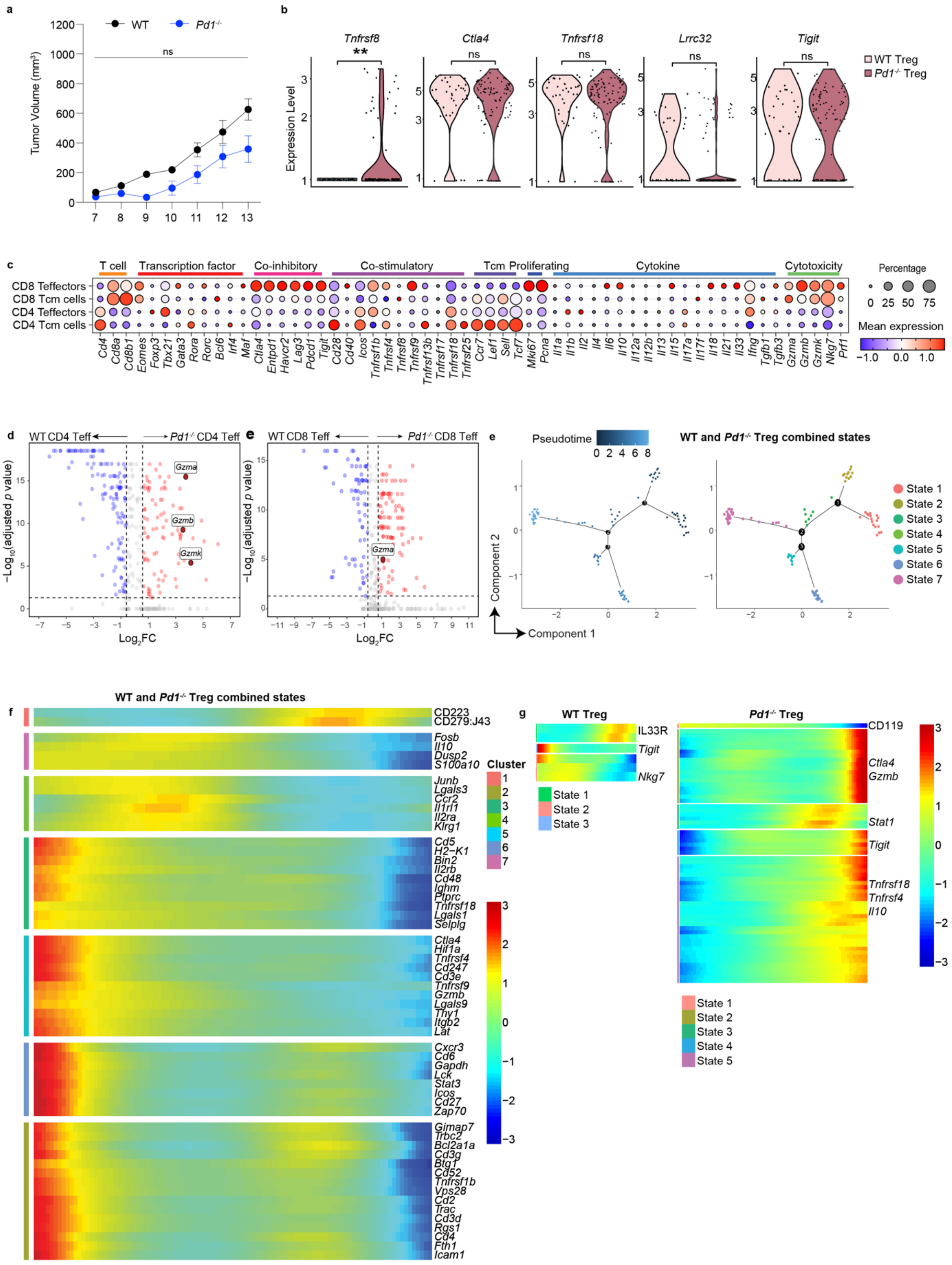

A, WT and *Pd1*$^{-/-}$ mice were inoculated subcutaneously with B16F10 melanoma and tumor growth was monitored. Tumors were harvested on day 14, TILs were subjected to BD

Rhapsody scRNA-seq analysis. **B**, Violin plot visualization of differences in *Tnfrsf8* (CD30)*, Ctla4, Tnfrsf18* (GITR)*, Lrrc32* (GARP) and *Tigit* transcript between Treg TILs from WT and $Pd1^{-/-}$ mice. **C**, Dot plot showing gene expression in 'T Effectors' subclusters. **D**, Volcano plot showing differential gene expression analysis of $CD4^{+}$ T effectors (Teff) and $CD8^{+}$ Teff. **E-G**, shows pseudotime and trajectory analysis performed using the monocle plugin in SeqGeq^{TM}. Data shown are from n=5 animals per cohort and each data point in **B** represents individual cells. Statistical analyses were performed using a two-way ANOVA with Sidak's multiple comparison in **A,** a two-tailed clustered Wilcoxon rank-sum test in **B** and a two-tailed Wilcoxon rank-sum test with Bonferroni correction in **D-E**. $^{**}P \leq 0.01$; ns, not significant.

Supplementary Figure 5. CellChat analysis of TIL immune cell signals, Related to Figure 2.

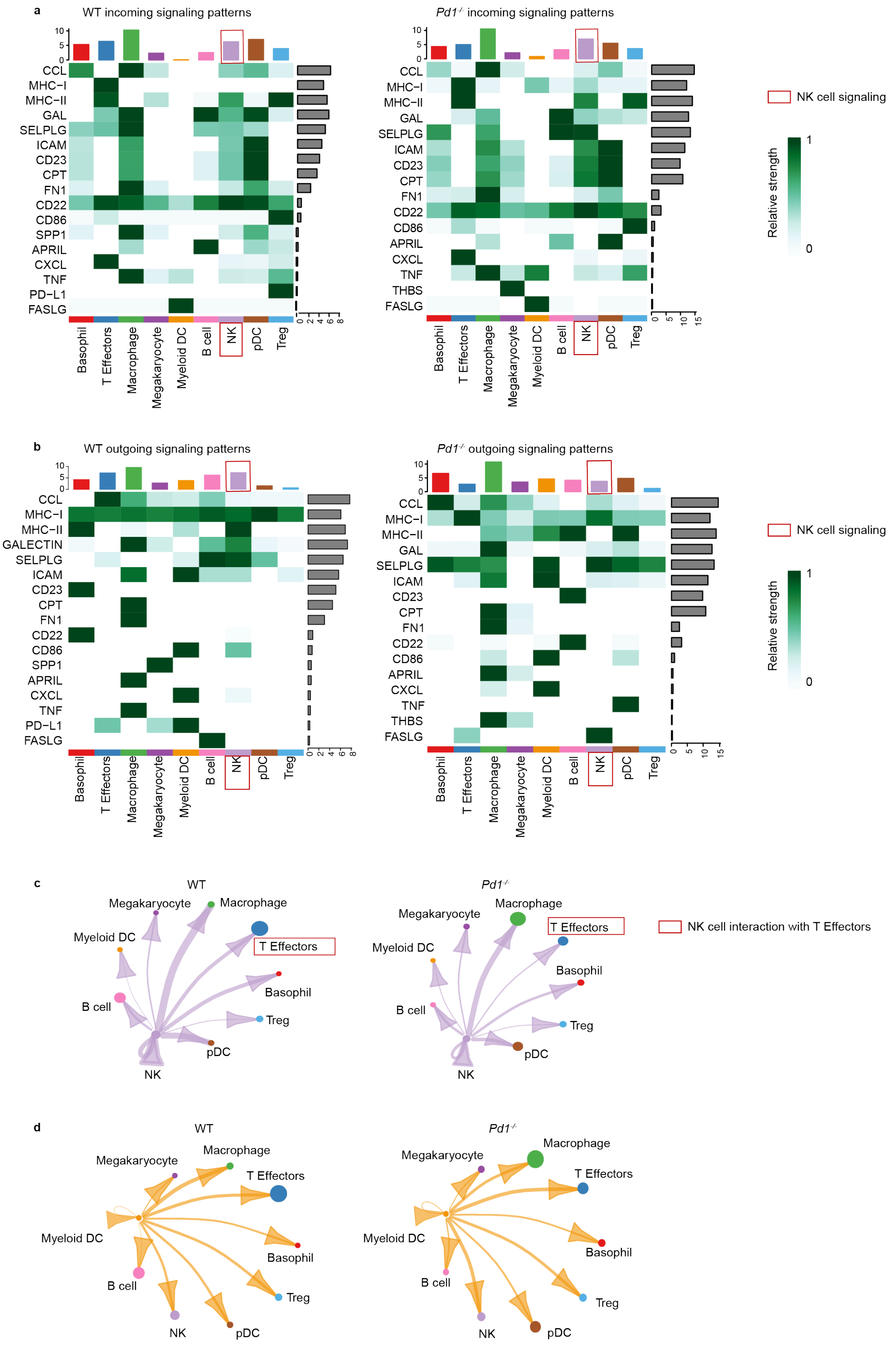

A-D, TILs isolated from B16F10-tumor bearing WT and *Pd1*$^{-/-}$ mice were subjected to BD Rhapsody sc-RNA seq analysis. CellChat analysis of signaling patterns of all immune cells in the TILs was analyzed. **A**, shows incoming signaling patterns in WT and *Pd1*$^{-/-}$ TILs, **B**, shows outgoing signaling patterns in WT and *Pd1*$^{-/-}$ TILs. **C-D**, Circle plot visualizing interactions of NK cells or myeloid DC towards TILs in WT and *Pd1*$^{-/-}$ cohort. Edge width (of lines) is proportional to the strength of interactions. Edges colored according to sending cell population. Data shown are from n=5 animals per cohort. CellChat models the communication probability based on the law of mass action and identifies significant communications using permutation tests.

Supplementary Figure 6. Immunobiology analysis of TILs to validate scRNA-seq dataset and CellChat predictive analysis, Related to Fig.3.

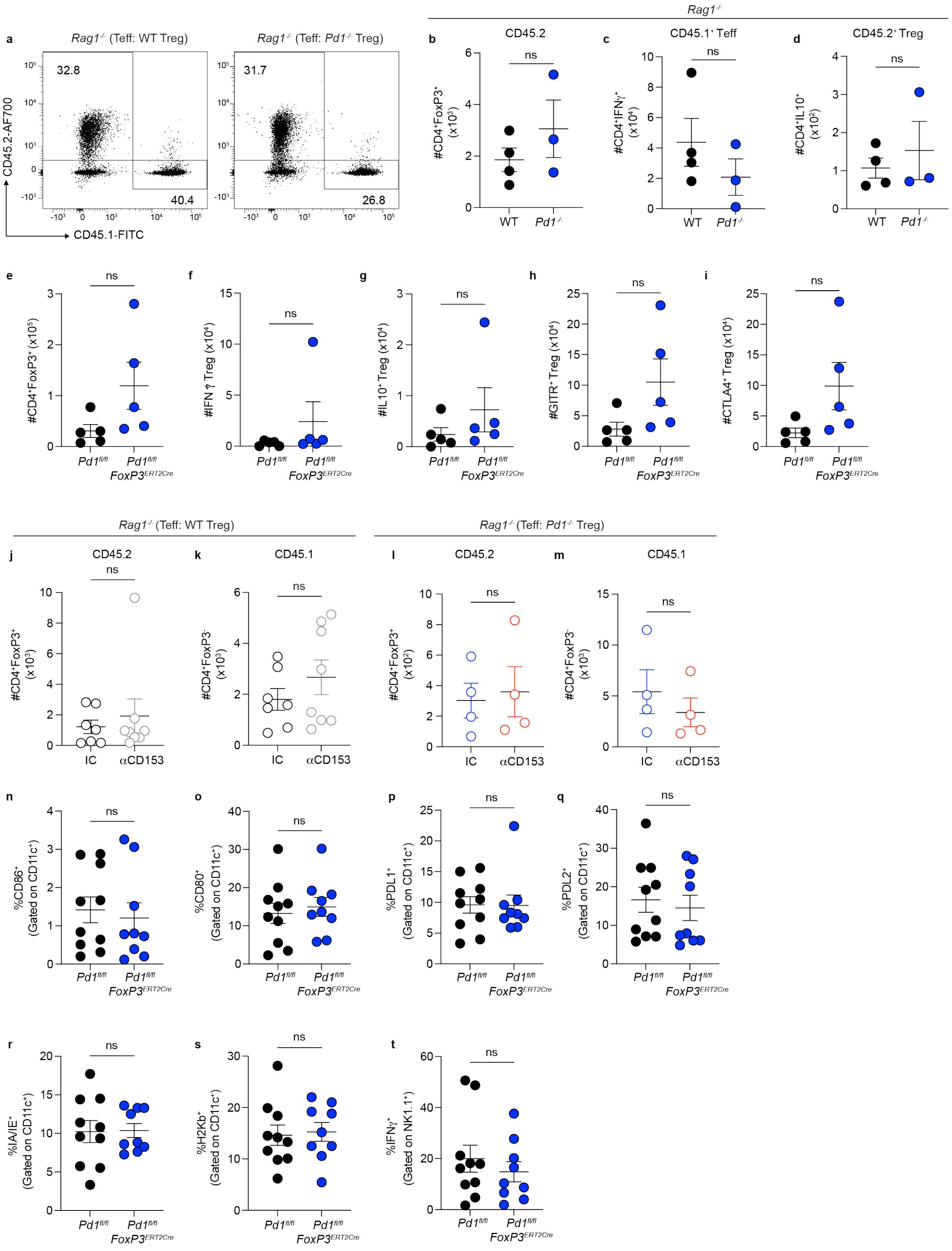

A-D, Tumor-bearing *Rag1*$^{-/-}$ mice were re-constituted with CD45.1 Teff cells along with CD45.2 Treg cells via IV injection at day 8 post inoculation. **A,** Representative flow cytometric plots of CD45.2 versus CD45.1 TILs. **B-D,** Absolute counts of CD45.2 Treg cells,

$CD45.1^+CD4^+Foxp3^-IFN\gamma^+$ and $CD45.2^+CD4^+Foxp3^+IL\text{-}10^+$ TILs (n=4 in Teff:WT Treg and n=3 in Teff:*Pd1*$^{-/-}$ Treg). **E-I,** *Pd1*$^{fl/fl}$ and *Pd1*$^{fl/fl}$*Foxp3*^{ERT2Cre} mice were treated with PBS or 1mg tamoxifen respectively for 5 consecutive dosages prior to B16F10 inoculation. **E-I**, Absolute count of Treg cells, IFNγ$^+$ Treg cells, IL10$^+$ Treg cells, GITR$^+$ Treg cells and CTLA4$^+$ Treg cells (n=5 per group). **J-M,** Tumor-bearing *Rag1*$^{-/-}$ mice were reconstituted with Teff cells along with either WT Treg cells or *Pd1*$^{-/-}$ Treg cells. Animals reconstituted with WT Treg cells or *Pd1*$^{-/-}$ Treg cells were treated with either isotype control or αCD153 for up to 5 dosages at two-days intervals. **J-K**, absolute count of CD45.2$^+$ Treg cells and CD45.1 $CD4^+Foxp3^-$ cells within the WT Treg treatment cohort is shown (n=7 IC vs n=8 αCD153). **L-M**, Absolute count of CD45.2 Treg cells and $CD45.1^+CD4^+Foxp3^-$ cells within the *Pd1*$^{-/-}$ Treg treatment cohort is shown (n=4 per group). **N-T**, TIL immunobiology in tumor reconstituted in *Pd1*$^{fl/fl}$ (n=10) and *Pd1*$^{fl/fl}$*Foxp3*^{ERT2Cre} (n=9) mice. **N-S**, Frequency of CD86, CD80, PDL1, PDL2, Class II expression and Class I expression in CD11c$^+$ DCs. **T**, IFNγ expression in NK1.1$^+$ cells. Data shown are mean±SEM with each data point representing an individual from at least three independent experiments. Statistical analyses were performed using a two-tailed unpaired Student's *t* test. ns, not significant.

Supplementary Figure 7. CosMx^{TM} analysis of B16 tumor tissue from *Rag1*$^{-/-}$ mice reconstituted with WT Teff along with either WT or *Pd1*$^{-/-}$ Treg, Related to Figure 5.

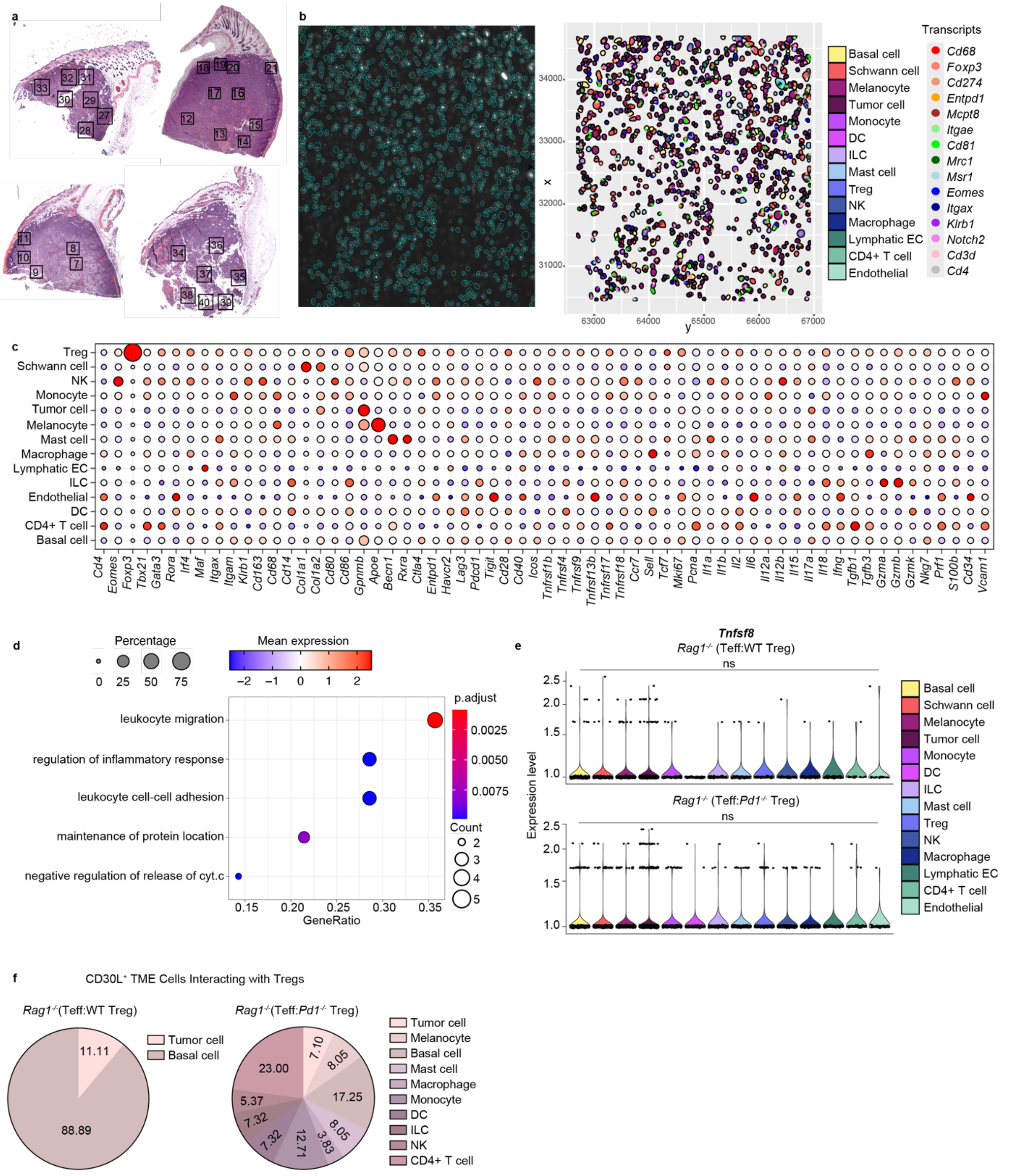

A-F, *Rag1*$^{-/-}$ mice were subcutaneously injected with B16F10 melanoma cells on d0 and further reconstituted with CD45.1^{+} Teff cells and CD45.2^{+} Treg cells sorted from either *Foxp3*^{RFP} (WT) or *Pd1*$^{-/-}$*Foxp3*^{RFP} (*Pd1*$^{-/-}$) mice via IV injection on d7. Tumors were harvested when tumor size reached 800mm^{3} and were subjected to CosMx^{TM} spatial transcriptomics analysis. Data are from n=2 *Rag1*$^{-/-}$ (WT Teff: WT Treg) and n=2 (WT Teff: *Pd1*$^{-/-}$ Treg) tumor tissue. **A,** H&E stain of FFPE punch biopsies derived from *Rag1*$^{-/-}$ mice reconstituted with WT Teff: WT Treg

(**left panel**) and WT Teff: *Pd1*$^{-/-}$ Treg (**right panel**), with field of views (FOVs) annotated. **B,** Cell segmentation (**left panel**) of representative FOV with cell identities and transcripts overlaid (**right panel**). **C**, Dot plot showing identification markers of all the clusters. **D**, shows pathways enriched in *Pd1*$^{-/-}$ Treg cells. **E,** Expression of *Tnfsf8* within the TME in *Rag1*$^{-/-}$ mice reconstituted with either WT or *Pd1*$^{-/-}$ Treg cells with CD4^{+} T eff cells. **F,** Pie chart showing frequency of *Tnfsf8*$^{+}$ TME cells interacting with WT or *Pd1*$^{-/-}$ Treg cells. Data shown represent individual cells in **E**. Statistical analyses were performed using a pairwise clustered Wilcoxon rank-sum test with FDR correction in **E**. ns, not significant.

Supplementary Figure 8: Cell cluster gene expression in human PBMCs and TILs from healthy control and melanoma patients subjected to scRNA-seq, Related to Figure 6.

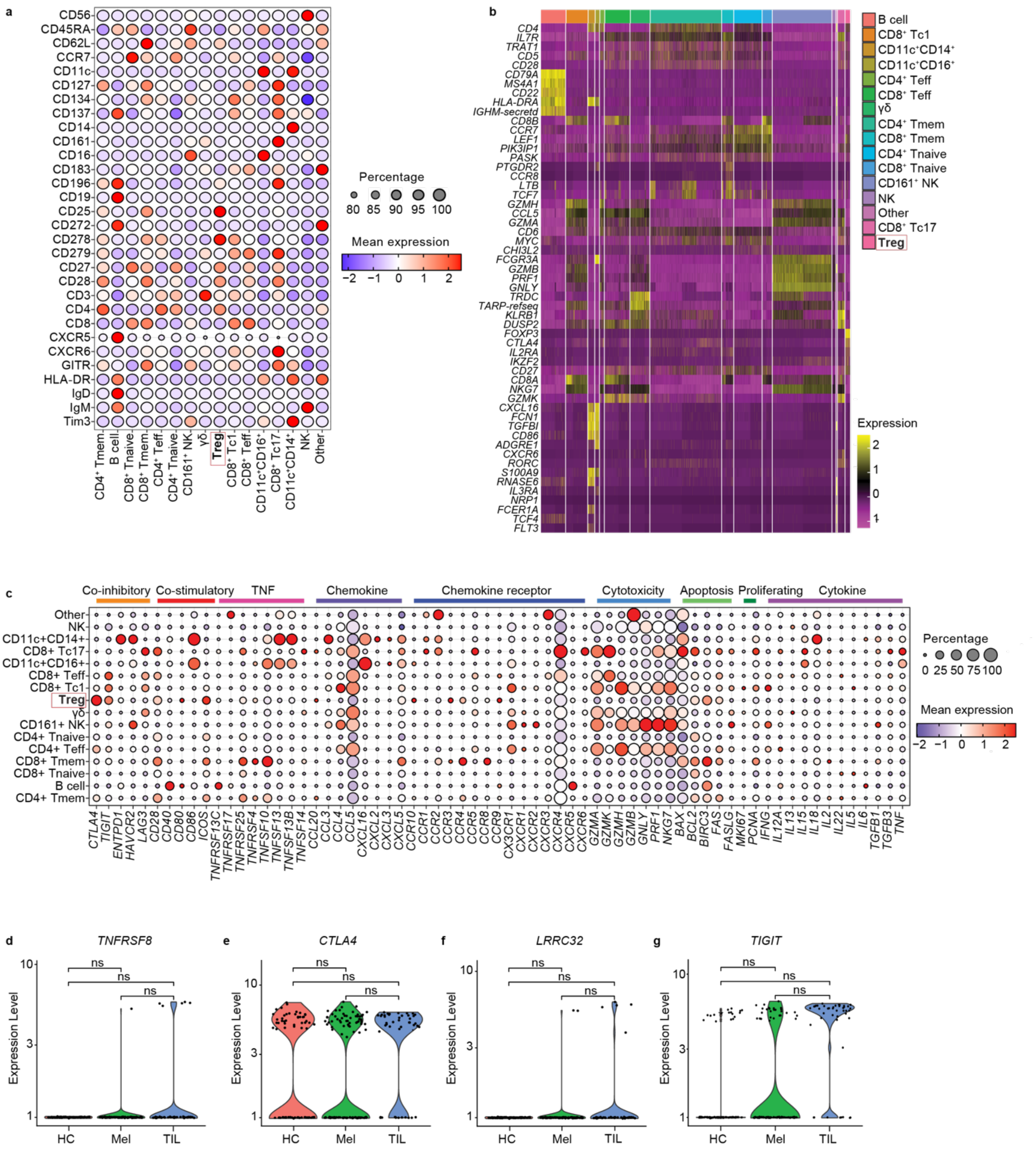

A-C, PBMCs from healthy controls (HC) and stage IV melanoma patients (Mel) were subjected to BD Rhapsody sc-RNA seq analysis. Data shown from 6042 cells in HC and 5592 cells in melanoma patient PBMCs. **A,** AbSeq protein expression for each cell type, where dot size and color represent percentage of protein expression and the averaged scaled expression value, respectively. **B,** Unbiased transcript analysis showing a heatmap of top 5 gene transcripts per cell type. **C**, Dot plot showing expression of candidate marker genes in all immune clusters. **D-**

G, Violin plots showing mRNA expression of *TNFRSF8* (CD30), *CTLA4, LRRC32* (GARP)*, TIGIT* in single cells. Data are derived from PBMCs of HC (n=3) and Mel (n=3), alongside TILs from treatment-naive melanoma patients (n=4). Data are representative of at least three independent experiments. Statistical analysis was performed using a pairwise clustered Wilcoxon rank-sum test with FDR correction in **D-G**. ns, not significant.

Supplementary Figure 9: Cell cluster gene expression in TILs from treatment-naive and anti-PD1 treated melanoma patients subjected to scRNA-seq, Related to Figure 6.

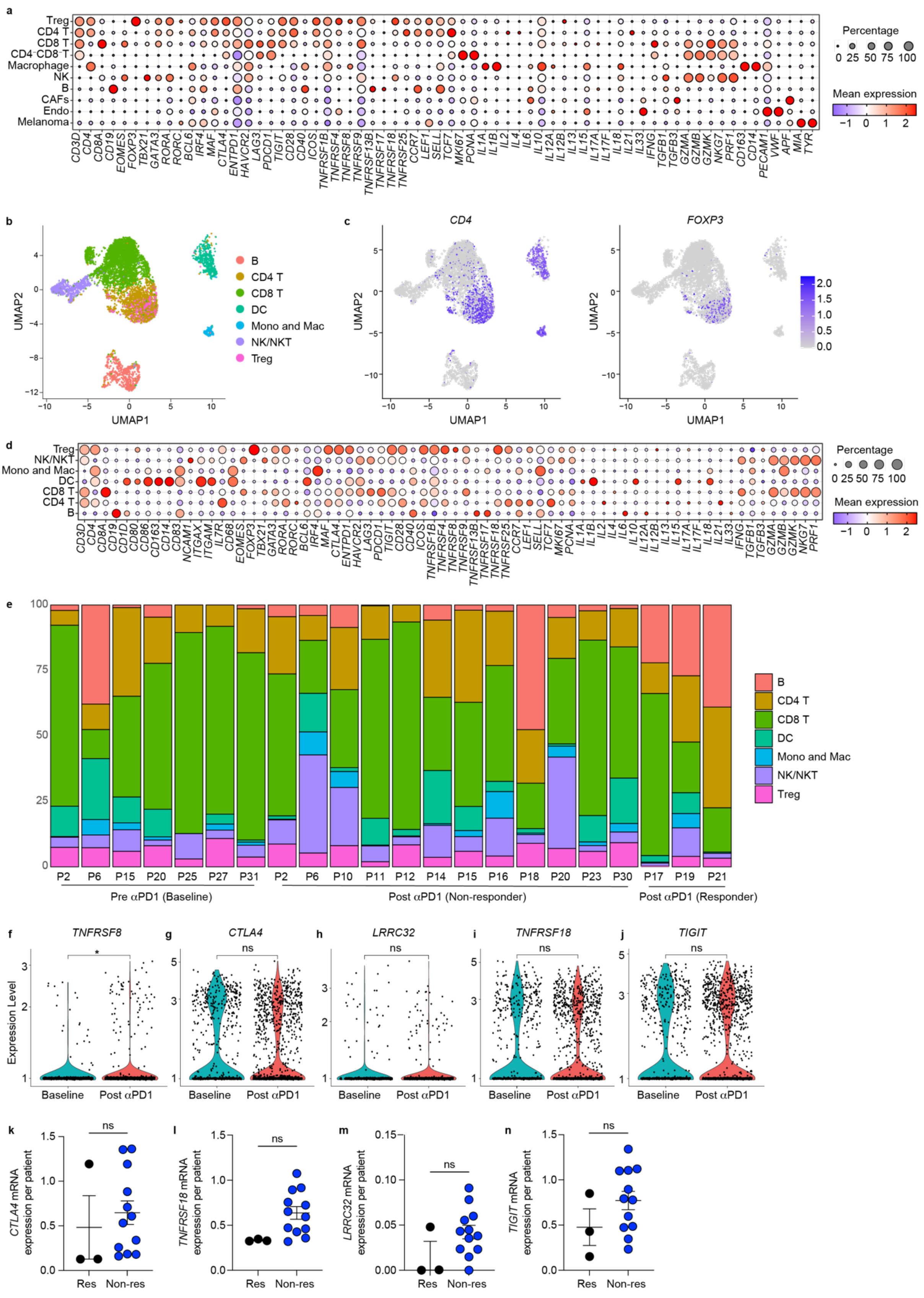

A, CD45^{+} and CD45^{-} tumors from naïve melanoma patient dataset was publicly mined and analyzed using RStudio. Gene expression within each cell cluster is shown as a dot plot. 1603 cells were used. **B**, Public dataset on TILs from melanoma patients at baseline or post anti-PD1 therapy was mined. Within this dataset, patients responding and non-responding to anti-PD1 were also present. 7564 cells were analyzed. Dataset was analyzed using RStudio. **B**, shows the UMAP. **C**, Feature plot showing *CD4* and *FOXP3* expression. **D**, Gene expression profile within each cluster as a dot plot. **E**, Frequency of cells within each cluster in individual patient. **F-J**, mRNA expression of *TNFRSF8* (CD30), *CTLA4, LRRC32* (GARP), *TNFRSF18* (GITR) and *TIGIT* in every single Treg cell (n=7 for baseline and n=12 for post anti-PD1 treatment). **K-N**, Mean mRNA expression of *CTLA4,* GARP, GITR and *TIGIT* in Treg cells from individual responders (n=3) and non-responders (n=12) is shown. Data shown represent individual cells in **F-J**. Data shown are mean±SEM, with each data point representing an individual patient in **K-N.** Data are representative of at least three independent experiments. A two-tailed clustered Wilcoxon rank-sum test was performed in **F-J** and a two-tailed unpaired Student's *t* test in **K-N**. ns, not significant.

Supplementary Table Titles

Supplementary Table 1. Detailed information on co-receptor combinations within boolean analysis in WT and *Pd1*$^{-/-}$ mice

Supplementary Table 2. Detailed information on co-receptor combinations within boolean analysis in WT and *Pd1*$^{fl/fl}$*Foxp3*^{ERT2Cre} mice

Supplementary Table 3. Detailed information of murine scRNA-seq analysis

Supplementary Table 4. Detailed information of murine CosMx™ spatial transcriptomic analysis

Supplementary Table 5. Detailed information of human scRNA-seq analysis
