# Supplementary material for: PD-1 receptor deficiency enhances CD30+ Treg cell function in melanoma
Source: Nat Immunol. 2025 Jun 2;26(7):1074–86. doi: 10.1038/s41590-025-02172-0 (PMC12208868; doi:10.1038/s41590-025-02172-0)
Supplement: Supplementary file 2 — Reporting Summary [file 41590_2025_2172_MOESM2_ESM.pdf]

nature portfolio

Corresponding author(s): Shoba Amarnath

Last updated by author(s): Apr 9, 2025

Reporting Summary

Nature Portfolio wishes to improve the reproducibility of the work that we publish. This form provides structure for consistency and transparency in reporting. For further information on Nature Portfolio policies, see our Editorial Policies and the Editorial Policy Checklist.

Statistics

For all statistical analyses, confirm that the following items are present in the figure legend, table legend, main text, or Methods section.

n/a	Confirmed	
☐	☒	The exact sample size (*n*) for each experimental group/condition, given as a discrete number and unit of measurement
☐	☒	A statement on whether measurements were taken from distinct samples or whether the same sample was measured repeatedly
☐	☒	The statistical test(s) used AND whether they are one- or two-sided<br>*Only common tests should be described solely by name; describe more complex techniques in the Methods section.*
☐	☒	A description of all covariates tested
☐	☒	A description of any assumptions or corrections, such as tests of normality and adjustment for multiple comparisons
☐	☒	A full description of the statistical parameters including central tendency (e.g. means) or other basic estimates (e.g. regression coefficient) AND variation (e.g. standard deviation) or associated estimates of uncertainty (e.g. confidence intervals)
☐	☒	For null hypothesis testing, the test statistic (e.g. *F*, *t*, *r*) with confidence intervals, effect sizes, degrees of freedom and *P* value noted<br>*Give P values as exact values whenever suitable.*
☒	☐	For Bayesian analysis, information on the choice of priors and Markov chain Monte Carlo settings
☒	☐	For hierarchical and complex designs, identification of the appropriate level for tests and full reporting of outcomes
☒	☐	Estimates of effect sizes (e.g. Cohen's *d*, Pearson's *r*), indicating how they were calculated

Our web collection on statistics for biologists contains articles on many of the points above.

Software and code

Policy information about availability of computer code

Data collection	BD LSRFortessa X-20 or BD FACSAria flow cytometers were used for flow cytometry data collection. LightCycler 480 II Instrument was used for RT-qPCR data collection. Both murine and human single-cell RNA sequencing were performed using the Illumina NovaSeq 6000. Zeiss AXIO Imager 2 was used for tumor H&E imaging. Morphology imaging and RNA readout in spatial transcriptomics were performed using the NanoString CosMx Spatial Molecular Imager instrument.
Data analysis	FlowJo software (v10.7.1) and SPICE (v6.1) were used for flow cytometry data analysis. Roche LightCycler 480 software was used to quantify RT-qPCR CT values. ScRNA-seq data collection was supported by BD Biosciences, with raw files uploaded to the Seven Bridges website, where quality control (QC) was performed by the BD Bioinformatics group. Spatial transcriptomics data was collected using the AtoMx Spatial Informatics Platform and exported via both the CosMx DA Export module and Flat Files Export module by NanoString at the Seattle Facility. SeqGeq software (v1.8) and RStudio (v4.4.1) were used for scRNA-seq analysis, while RStudio (v4.4.1) was used exclusively for spatial transcriptomics analysis. The Seurat R package (v5.1.0) was used to normalize counts, perform QC, generate UMAP and violin plots, visualize field-of-view (FOV), and conduct differential gene expression analysis. The ggplot2 R package (v3.5.1) was used to visualize cell-type frequencies as a bar chart and generate volcano plots. Gene expression visualization as a dot plot was done using the scRNAtoolVis R package (v0.1.0). Ensembl data was accessed through the biomaRt R package (v2.60.1) to obtain Entrez Gene IDs for murine datasets. The org.mm.eg.db (v3.19.1) R packages were used for murine gene annotation. Gene Ontology (GO) analysis was performed using the enrichplot R package (v3.4), while Gene Set Enrichment Analysis (GSEA) was performed using the clusterProfiler R package (v4.12.6). Pseudotime trajectory analysis was performed using the monocle R package (v2.32.0) in both SeqGeq software and RStudio. Cell-cell communication analysis was performed using the CellChat R package (v1.6.1). Statistical analysis was conducted using GraphPad Prism (v10.4.0), Microsoft Excel (v16.91), R base, and the clusrank R package (v1.0-4).

For manuscripts utilizing custom algorithms or software that are central to the research but not yet described in published literature, software must be made available to editors and reviewers. We strongly encourage code deposition in a community repository (e.g. GitHub). See the Nature Portfolio guidelines for submitting code & software for further information.

Data

Policy information about availability of data

All manuscripts must include a data availability statement. This statement should provide the following information, where applicable:

- Accession codes, unique identifiers, or web links for publicly available datasets
- A description of any restrictions on data availability
- For clinical datasets or third party data, please ensure that the statement adheres to our policy

The CHIP-seq dataset and STAT5 RNA-seq dataset were taken from previously published work and it is available through the Gene Expression Omnibus (GEO) under accession number GSE207265. The BD Rhapsody scRNA-seq for murine and human datasets are available at GEO under accession number GSE273532 and GSE280319 respectively. TThe NanoString CosMx spatial transcriptomics datasets are available at GEO under accession number GSE273530. The publicly mined datasets for human melanoma samples are available at GEO under accession number GSE72056 and GSE120575. The datasets used in CD30 biomarker analysis is available at TCGA and GEO under accession number GSE42568 and GSE33114. CellChat database for murine datasets utilized for cell-cell communication analysis is available at https://github.com/sqjin/CellChat.

Research involving human participants, their data, or biological material

Policy information about studies with human participants or human data. See also policy information about sex, gender (identity/presentation), and sexual orientation and race, ethnicity and racism.

Reporting on sex and gender	Sex and gender does not affect the current study.
Reporting on race, ethnicity, or other socially relevant groupings	not applicable as only the biological material was used.
Population characteristics	No data on population characteristics was collected.
Recruitment	The patients and healthy controls used in this study were recruited as follows: Healthy control leukapheresis products were obtained from the Newcastle Upon Tyne hospitals Blood Bank under approved ethics. The patients were recrutied by Dr. Ioana Cosagera after informed consent. Patients who provided consent were recruited. There was no self-selection or other biasis in recruitment.
Ethics oversight	Royal Victoria Infirmary in Newcastle upon Tyne under ethics [REC reference 24/NE/0014]

Note that full information on the approval of the study protocol must also be provided in the manuscript.

Field-specific reporting

Please select the one below that is the best fit for your research. If you are not sure, read the appropriate sections before making your selection.

☒ Life sciences ☐ Behavioural & social sciences ☐ Ecological, evolutionary & environmental sciences

For a reference copy of the document with all sections, see nature.com/documents/nr-reporting-summary-flat.pdf

Life sciences study design

All studies must disclose on these points even when the disclosure is negative.

Sample size	Sample size were calculated for in-vivo and ex-vivo analysis as follows. All power calculations are based on Rosner B, Fundamentals in Biostatistics, F2010:301-307. In order to obtain a difference from an independent control and experimental mice with 1 control(s) per experimental mice, with normal distribution n=5-7 mice would be required to reject the null hypothesis with probability(power) 0.8. The Type 1 error probability associated with this test of null hypothesis is 0.05. For in-vitro experiments, at least three replicates were performed
Data exclusions	Data points were not excluded in this study.
Replication	All experiments involving in-vitro cell culture were carried out atleast three times. For ex-vivo analysis, atleast n=5 mice were used namely immunophenotyping studies. For in-vivo studies n=3-5 mice were used per experiment and experiments were repeated twice. All replicates were successful.
Randomization	Randomization was utilized for tumor experiments when the animals were reconstituted with immune cells. Here, tumor size and sex was matched on day 5 or 7 and then WT or KO immune cells were injected. For in-vitro analysis, mice at 8 weeks old were used and this was not randomized.
Blinding	Animals within the in-vivo tumor protocols were monitored for weight loss in a blinded fashion by the animal technicians while they were in study. Blinding was not necessary for tumor measurement experiments since only objective measurable criteria were used for this study.

Reporting for specific materials, systems and methods

We require information from authors about some types of materials, experimental systems and methods used in many studies. Here, indicate whether each material, system or method listed is relevant to your study. If you are not sure if a list item applies to your research, read the appropriate section before selecting a response.

Materials & experimental systems

n/a	Involved in the study
☐	☒ Antibodies
☐	☒ Eukaryotic cell lines
☒	☐ Palaeontology and archaeology
☐	☒ Animals and other organisms
☒	☐ Clinical data
☒	☐ Dual use research of concern
☒	☐ Plants

Methods

n/a	Involved in the study
☒	☐ ChIP-seq
☐	☒ Flow cytometry
☒	☐ MRI-based neuroimaging

Antibodies

Antibodies used

Immunophenotyping antibodies:
Anti-mouse/human B220-Biotin, RA3-6B2, 103204, BioLegend, 1:200
Anti-mouse Bcl2-FITC, 10C4, 11-6992-41, eBioscience, 1:200
Anti-mouse CD119-PE, 2E2, 12-1191-82, eBioscience, 1:200
Anti-mouse CD11b-Biotin, N418, 117304, BioLegend, 1:200
Anti-mouse CD11b-PE, N418, 117307, BioLegend, 1:200
Anti-mouse CD152-BV605, UC10-4B9, 106323, BioLegend, 1:200
Anti-mouse CD19-Biotin, 6D5, 115504, BioLegend, 1:200
Anti-mouse CD3ε-Biotin, 145-2C1, 100303, Biolegend, 1:200
Anti-mouse CD25-FITC, PC61, 102005, BioLegend, 1:200
Anti-mouse CD25-BV785, PC61, 102051, BioLegend, 1:200
Anti-mouse CD30-Biotin, mCD30.1, 102303, BioLegend, 1:200
Anti-mouse CD30-PE, mCD30.1, 102306, BioLegend, 1:200
Anti-mouse CD4-Biotin, GK.15, 100404, BioLegend, 1:200
Anti-mouse CD4-AF700, GK1.5, 100429, BioLegend, 1:200
Anti-mouse CD4-BV510, GK1.5, 100449, BioLegend, 1:200
Anti-mouse CD4-BV711, GK1.5, 100447, BioLegend, 1:200
Anti-mouse/human CD44-APC/Cy7, IM7, 103027, BioLegend, 1:200
Anti-mouse/human CD44-AF700, IM7, 103025, BioLegend, 1:200
Anti-mouse CD45.1-FITC, A20, 110705, BioLegend, 1:200
Anti-mouse CD45.2-AF700, 104, 109821, BioLegend, 1:200
Anti-mouse CD49b-Biotin, DX5, 108904, BioLegend, 1:200
Anti-mouse CD5-Biotin, 53-7.3, 100604, BioLegend, 1:200
Anti-mouse CD62L-BV510, MEL-14, 104441, BioLegend, 1:200
Anti-mouse CD62L-BV650, MEL-14, 104453, BioLegend, 1:200
Anti-mouse CD8α-Biotin, 53-6.7, 100704, BioLegend, 1:200
Anti-mouse CD80-BV421, 16-10A1, 104725, BioLegend, 1:200
Anti-mouse CD86-AF700, A17199A, 159213, BioLegend, 1:200
Anti-mouse CD90.2-AF700, 30-H12, 105320, BioLegend, 1:400
Anti-mouse F4/80-Biotin, BM8, 123106, BioLegend, 1:200
Anti-mouse Foxp3-APC, FJK-16s, 17-5773-80, eBioscience, 1:200
Anti-mouse Foxp3-PE, FJK-16s, 12-5773-80, eBioscience, 1:200
Anti-mouse Foxp3-AF700, FJK-16s, 56-5773-82, eBioscience, 1:200
Anti-mouse Foxp3-FITC, FJK-16s, 11-5773-82, eBioscience, 1:200
Anti-mouse GARP-PECy7, YGIC86, 25-9891-82, eBioscience, 1:200
Anti-mouse GATA3-PECy7, TWAJ, 25-9966-42, eBioscience, 1:200
Anti-mouse GITR-BV786, DTA-1, 741020, BD Biosciences, 1:200
Anti-mouse Gr1-Biotin, RB6-8C5, 108404, BioLegend, 1:200
Anti-mouse/human GzmB-PerCP/Cy5.5, QA16A02, 372211, BioLegend, 1:100
Anti-mouse H-2Kb-BV510, AF6-88.5, 116523, BioLegend, 1:200
Anti-mouse/human Helios-AF700, 22F6, 137241, BioLegend, 1:200
Anti-mouse I-A/I-E-APC, M5/114.15.2, 107613, BioLegend, 1:200
Anti-mouse IFN-γ-BV510, XMG1.2, 505841, BioLegend, 1:200
Anti-mouse IL-10-BV605, JES5-16E3, 505031, BioLegend, 1:200
Anti-mouse IL-10-PE, JES5-16E3, 505007, BioLegend, 1:200
Anti-mouse IL-17A-APC/Cy7, TC11-18H10.1, 506939, BioLegend, 1:200
Anti-mouse/human Ki67-FITC, SolA15, 11-5698-80, eBioscience, 1:200
Anti-mouse NK1.1-FITC, PK136, 108706, BioLegend, 1:200
Anti-mouse Nrp1-APC, 3E12, 145205, BioLegend, 1:200
Anti-mouse PD-1-APC/Cy7, 29F.1A12, 135223, BioLegend, 1:200

Anti-mouse PD-L1-BV785, 10F.9G2, 124331, BioLegend, 1:200
Anti-mouse PD-L2-PE/Dazz594, TY25, 107215, BioLegend, 1:200
Anti-mouse pSTAT5-AF488, 47, 612598, BD Biosciences, 1:5
Anti-mouse/human RORγt-PE, B2D, 12-6981-80, eBioscience, 1:150
Anti-mouse ST2-PE, DJ8, 101001PE, MD Bioproducts, 1:200
Anti-mouse T-bet-FITC, 4B10, 644811, BioLegend, 1:100
Anti-mouse Ter119-Biotin, TER-119, 116204, BioLegend, 1:200
Anti-mouse TIGIT-BV421, 1G9, 142111, BioLegend, 1:200
Anti-mouse Thy1-AF700, 30-H12, 109007, BioLegend, 1:200
Anti-mouse TruStain FcX™, 93, 101320, BioLegend, 1:100
Anti-human CD4-AF700, RPA-T4, 300526, BioLegend, 1:200
Anti-human Foxp3-BV421, 206D, 320123, BioLegend, 1:200
Anti-human GITR-APC/Fire750, 108-17, 371221, BioLegend, 1:200
Anti-human GARP-PerCP/Cy5.5, 7B11, 352513, BioLegend, 1:200
Anti-human TIGIT-BV510, A15153G, 372737, BioLegend, 1:200
Anti-human CD30-APC, BY88, 333909, BioLegend, 1:200
Anti-human CD152-BV711, BNI3, 369631, BioLegend, 1:200
Anti-human CD45-PE, HI30, 304007, BioLegend, 1:200
Anti-human TruStain FcX™, 422301, BioLegend, 1:100
Anti-Annexin V-PE, 556421, BD Biosciences, 1:20

In Vitro Antibodies:
Anti-mouse CD3-Ultra-LEAF Purified, 145-2C11, 100339, BioLegend, 5µg/ml
Anti-mouse CD28-Ultra-LEAF Purified, 37.51, 102115, BioLegend, 2µg/ml
Anti-mouse IL-2-Functional Grade, S4B6, 16-7020-85, Invitrogen, 10µg/ml
Anti-mouse PD-1-InVivoMAb, RMP1.14, BE0146, Bio X Cell, 10µg/ml
Anti-human CD3-Ultra-LEAF Purified, OKT3, 317325, BioLegend, 0.5µg/ml
Anti-human PD-L1-Ultra-LEAF Purified,, 29E.2A3, 329715, BioLegend, 20µg/ml
Mouse Isotype Control IgG2b-Ultra-LEAF Purified, MPC-11, 400347, Biolegend, 20µg/ml

In Vivo Antibodies:
Anti-mouse CD153, RM153, AB01089-2-0-VXM, 2BScientific, 0.1mg/dose
Rat Isotype control IgG2a-InVivoMAb, 2A3, BE0089, Bio X Cell, 0.1mg/dose

Validation

All the antibodies used were standard commercial antibodies. Validation statement were confirmed from the manufacturer's website for their relevant used in the study.

Anti-mouse/human B220-Biotin, RA3-6B2, 103204, BioLegend, 1:200
https://www.biolegend.com/en-gb/products/biotin-anti-mouse-human-cd45r-b220-antibody-444
Anti-mouse Bcl2-FITC, 10C4, 11-6992-41, eBioscience, 1:200
https://www.thermofisher.com/antibody/product/Bcl-2-Antibody-clone-10C4-Monoclonal/11-6992-42
Anti-mouse CD119-PE, 2E2, 12-1191-82, eBioscience, 1:200
https://www.thermofisher.com/antibody/product/CD119-IFN-gamma-Receptor-1-Antibody-clone-2E2-Monoclonal/12-1191-82
Anti-mouse CD11b-Biotin, N418, 117304, BioLegend, 1:200
https://www.biolegend.com/en-gb/products/biotin-anti-mouse-cd11c-antibody-1814
Anti-mouse CD11b-PE, N418, 117307, BioLegend, 1:200
https://www.biolegend.com/en-gb/products/pe-anti-mouse-cd11c-antibody-1816
Anti-mouse CD152-BV605, UC10-4B9, 106323, BioLegend, 1:200
https://www.biolegend.com/en-gb/products/brilliant-violet-605-anti-mouse-cd152-antibody-12375
Anti-mouse CD19-Biotin, 6D5, 115504, BioLegend, 1:200
https://www.biolegend.com/en-gb/products/biotin-anti-mouse-cd19-antibody-1527
Anti-mouse CD3ε-Biotin, 145-2C1, 100303, Biolegend, 1:200
https://www.biolegend.com/en-gb/products/biotin-anti-mouse-cd3epsilon-antibody-22
Anti-mouse CD25-FITC, PC61, 102005, BioLegend, 1:200
https://www.biolegend.com/en-gb/products/fitc-anti-mouse-cd25-antibody-422
Anti-mouse CD25-BV785, PC61, 102051, BioLegend, 1:200
https://www.biolegend.com/en-gb/products/brilliant-violet-785-anti-mouse-cd25-antibody-10293
Anti-mouse CD30-Biotin, mCD30.1, 102303, BioLegend, 1:200
https://www.biolegend.com/en-gb/products/biotin-anti-mouse-cd30-antibody-527
Anti-mouse CD30-PE, mCD30.1, 102306, BioLegend, 1:200
https://www.biolegend.com/en-gb/products/pe-anti-mouse-cd30-antibody-529
Anti-mouse CD4-Biotin, GK.15, 100404, BioLegend, 1:200
https://www.biolegend.com/en-gb/products/biotin-anti-mouse-cd4-antibody-247
Anti-mouse CD4-AF700, GK1.5, 100429, BioLegend, 1:200
https://www.biolegend.com/en-gb/products/alexa-fluor-700-anti-mouse-cd4-antibody-3385
Anti-mouse CD4-BV510, GK1.5, 100449, BioLegend, 1:200
https://www.biolegend.com/en-gb/products/brilliant-violet-510-anti-mouse-cd4-antibody-10707
Anti-mouse CD4-BV711, GK1.5, 100447, BioLegend, 1:200
https://www.biolegend.com/en-gb/products/brilliant-violet-711-anti-mouse-cd4-antibody-10706
Anti-mouse/human CD44-APC/Cy7, IM7, 103027, BioLegend, 1:200
https://www.biolegend.com/en-gb/products/apc-cyanine7-anti-mouse-human-cd44-antibody-3933
Anti-mouse/human CD44-AF700, IM7, 103025, BioLegend, 1:200
https://www.biolegend.com/en-gb/products/alexa-fluor-700-anti-mouse-human-cd44-antibody-3406
Anti-mouse CD45.1-FITC, A20, 110705, BioLegend, 1:200
https://www.biolegend.com/en-gb/products/fitc-anti-mouse-cd45-1-antibody-198
Anti-mouse CD45.2-AF700, 104, 109821, BioLegend, 1:200

https://www.biolegend.com/en-gb/products/alexa-fluor-700-anti-mouse-cd45-2-antibody-3393
Anti-mouse CD49b-Biotin, DX5, 108904, BioLegend, 1:200
https://www.biolegend.com/en-gb/products/biotin-anti-mouse-cd49b-pan-nk-cells-antibody-232
Anti-mouse CD5-Biotin, 53-7.3, 100604, BioLegend, 1:200
https://www.biolegend.com/en-gb/products/biotin-anti-mouse-cd5-antibody-158
Anti-mouse CD62L-BV510, MEL-14, 104441, BioLegend, 1:200
https://www.biolegend.com/en-gb/products/brilliant-violet-510-anti-mouse-cd62l-antibody-8162
Anti-mouse CD62L-BV650, MEL-14, 104453, BioLegend, 1:200
https://www.biolegend.com/en-gb/products/brilliant-violet-650-anti-mouse-cd62l-antibody-17377
Anti-mouse CD8α-Biotin, 53-6.7, 100704, BioLegend, 1:200
https://www.biolegend.com/en-gb/products/biotin-anti-mouse-cd8a-antibody-152
Anti-mouse CD80-BV421, 16-10A1, 104725, BioLegend, 1:200
https://www.biolegend.com/en-gb/products/brilliant-violet-421-anti-mouse-cd80-antibody-7357
Anti-mouse CD86-AF700, A17199A, 159213, BioLegend, 1:200
https://www.biolegend.com/en-gb/products/alexa-fluor-700-anti-mouse-cd86-antibody-24671
Anti-mouse CD90.2-AF700, 30-H12, 105320, BioLegend, 1:400
https://www.biolegend.com/en-gb/products/alexa-fluor-700-anti-mouse-cd90-2-thy1-2-antibody-3412
Anti-mouse F4/80-Biotin, BM8, 123106, BioLegend, 1:200
https://www.biolegend.com/en-gb/products/biotin-anti-mouse-f4-80-antibody-4066
Anti-mouse Foxp3-APC, FJK-16s, 17-5773-80, eBioscience, 1:200
https://www.thermofisher.com/antibody/product/FOXP3-Antibody-clone-FJK-16s-Monoclonal/17-5773-80
Anti-mouse Foxp3-PE, FJK-16s, 12-5773-80, eBioscience, 1:200
https://www.thermofisher.com/antibody/product/FOXP3-Antibody-clone-FJK-16s-Monoclonal/12-5773-80
Anti-mouse Foxp3-AF700, FJK-16s, 56-5773-82, eBioscience, 1:200
https://www.thermofisher.com/antibody/product/FOXP3-Antibody-clone-FJK-16s-Monoclonal/56-5773-82
Anti-mouse Foxp3-FITC, FJK-16s, 11-5773-82, eBioscience, 1:200
https://www.thermofisher.com/antibody/product/FOXP3-Antibody-clone-FJK-16s-Monoclonal/11-5773-82
Anti-mouse GARP-PECy7, YGIC86, 25-9891-82, eBioscience, 1:200
https://www.thermofisher.com/antibody/product/GARP-Antibody-clone-YGIC86-Monoclonal/25-9891-82
Anti-mouse GATA3-PECy7, TWAJ, 25-9966-42, eBioscience, 1:200
https://www.thermofisher.com/antibody/product/Gata-3-Antibody-clone-TWAJ-Monoclonal/25-9966-42
Anti-mouse GITR-BV786, DTA-1, 741020, BD Biosciences, 1:200
https://www.bdbiosciences.com/en-eu/products/reagents/flow-cytometry-reagents/research-reagents/single-color-antibodies-ruo/bv786-rat-anti-mouse-cd357-gitr.741020?tab=product_details
Anti-mouse Gr1-Biotin, RB6-8C5, 108404, BioLegend, 1:200
https://www.biolegend.com/en-gb/products/biotin-anti-mouse-ly-6g-ly-6c-gr-1-antibody-457
Anti-mouse/human GzmB-PerCP/Cy5.5, QA16A02, 372211, BioLegend, 1:100
https://www.biolegend.com/en-gb/products/percp-cyanine5-5-anti-humanmouse-granzyme-b-recombinant-antibody-15597
Anti-mouse H-2Kb-BV510, AF6-88.5, 116523, BioLegend, 1:200
https://www.biolegend.com/en-gb/products/brilliant-violet-510-anti-mouse-h-2k-b-antibody-17361
Anti-mouse/human Helios-AF700, 22F6, 137241, BioLegend, 1:200
https://www.biolegend.com/en-gb/products/alexa-fluor-700-anti-mouse-human-helios-antibody-18930
Anti-mouse I-A/I-E-APC, M5/114.15.2, 107613, BioLegend, 1:200
https://www.biolegend.com/en-gb/products/apc-anti-mouse-i-a-i-e-antibody-2488
Anti-mouse IFN-γ-BV510, XMG1.2, 505841, BioLegend, 1:200
https://www.biolegend.com/en-gb/products/brilliant-violet-510-anti-mouse-ifn-gamma-antibody-8610
Anti-mouse IL-10-BV605, JES5-16E3, 505031, BioLegend, 1:200
https://www.biolegend.com/en-gb/products/brilliant-violet-605-anti-mouse-il-10-antibody-9382
Anti-mouse IL-10-PE, JES5-16E3, 505007, BioLegend, 1:200
https://www.biolegend.com/en-gb/products/pe-anti-mouse-il-10-antibody-944
Anti-mouse IL-17A-APC/Cy7, TC11-18H10.1, 506939, BioLegend, 1:200
https://www.biolegend.com/en-gb/products/apc-cyanine7-anti-mouse-il-17a-antibody-11968
Anti-mouse/human Ki67-FITC, SolA15, 11-5698-80, eBioscience, 1:200
https://www.thermofisher.com/antibody/product/Ki-67-Antibody-clone-SolA15-Monoclonal/11-5698-80
Anti-mouse NK1.1-FITC, PK136, 108706, BioLegend, 1:200
https://www.biolegend.com/en-gb/products/fitc-anti-mouse-nk-1-1-antibody-429
Anti-mouse Nrp1-APC, 3E12, 145205, BioLegend, 1:200
https://www.biolegend.com/en-gb/products/apc-anti-mouse-cd304-neuropilin-1-antibody-8506
Anti-mouse PD-1-APC/Cy7, 29F.1A12, 135223, BioLegend, 1:200
https://www.biolegend.com/en-gb/products/apc-cyanine7-anti-mouse-cd279-pd-1-antibody-9742
Anti-mouse PD-L1-BV785, 10F.9G2, 124331, BioLegend, 1:200
https://www.biolegend.com/en-gb/products/brilliant-violet-785-anti-mouse-cd274-b7-h1-pd-l1-antibody-13497
Anti-mouse PD-L2-PE/Dazz594, TY25, 107215, BioLegend, 1:200
https://www.biolegend.com/en-gb/products/pedazzle594-anti-mouse-cd273-antibody-15630
Anti-mouse pSTAT5-AF488, 47, 612598, BD Biosciences, 1:5
https://www.bdbiosciences.com/en-eu/products/reagents/flow-cytometry-reagents/research-reagents/single-color-antibodies-ruo/alexa-fluor-488-anti-stat5-py694.612598?tab=product_details
Anti-mouse/human RORγt-PE, B2D, 12-6981-80, eBioscience, 1:150
https://www.thermofisher.com/antibody/product/ROR-gamma-t-Antibody-clone-B2D-Monoclonal/12-6981-80
Anti-mouse ST2-PE, DJ8, 101001PE, MD Bioproducts, 1:200
https://www.mdbioproducts.com/collections/antibodies/products/t1-st2-il-33-r-mouse-monoclonal-antibody-pe-conjugated?variant=39848199815357
Anti-mouse T-bet-FITC, 4B10, 644811, BioLegend, 1:100
https://www.biolegend.com/en-gb/products/fitc-anti-t-bet-antibody-6435
Anti-mouse Ter119-Biotin, TER-119, 116204, BioLegend, 1:200
https://www.biolegend.com/en-gb/products/biotin-anti-mouse-ter-119-erythroid-cells-antibody-1864

Anti-mouse TIGIT-BV421, 1G9, 142111, BioLegend, 1:200
https://www.biolegend.com/en-gb/products/brilliant-violet-421-anti-mouse-tigit-vstm3-antibody-14595
Anti-mouse Thy1-AF700, 30-H12, 109007, BioLegend, 1:200
https://www.biolegend.com/en-gb/products/alexa-fluor-700-anti-mouse-cd90-2-thy1-2-antibody-3412
Anti-mouse TruStain FcX™, 93, 101320, BioLegend, 1:100
https://www.biolegend.com/en-gb/products/trustain-fcx-anti-mouse-cd16-32-antibody-5683
Anti-human CD4-AF700, RPA-T4, 300526, BioLegend, 1:200
https://www.biolegend.com/en-gb/products/alexa-fluor-700-anti-human-cd4-antibody-3395
Anti-human Foxp3-BV421, 206D, 320123, BioLegend, 1:200
https://www.biolegend.com/en-gb/products/brilliant-violet-421-anti-human-foxp3-antibody-12045
Anti-human GITR-APC/Fire750, 108-17, 371221, BioLegend, 1:200
https://www.biolegend.com/en-gb/products/apc-fire-750-anti-human-cd357-gitr-antibody-14487
Anti-human GARP-PerCP/Cy5.5, 7B11, 352513, BioLegend, 1:200
https://www.biolegend.com/en-gb/products/percp-cyanine5-5-anti-garp-lrrc32-antibody-15750
Anti-human TIGIT-BV510, A15153G, 372737, BioLegend, 1:200
https://www.biolegend.com/en-gb/products/brilliant-violet-510-anti-human-tigit-vstm3-antibody-19940
Anti-human CD30-APC, BY88, 333909, BioLegend, 1:200
https://www.biolegend.com/en-gb/products/apc-anti-human-cd30-antibody-5018
Anti-human CD152-BV711, BNI3, 369631, BioLegend, 1:200
https://www.biolegend.com/en-gb/products/brilliant-violet-711-anti-human-cd152-ctla-4-antibody-20733
Anti-human CD45-PE, HI30, 304007, BioLegend, 1:200
https://www.biolegend.com/en-gb/products/pe-anti-human-cd45-antibody-708
Anti-human TruStain FcX™, 422301, BioLegend, 1:100
https://www.biolegend.com/en-gb/products/human-trustain-fcx-fc-receptor-blocking-solution-6462
Anti-Annexin V-PE, 556421, BD Biosciences, 1:20
https://www.bdbiosciences.com/en-eu/products/reagents/flow-cytometry-reagents/research-reagents/single-color-antibodies-ruo/pe-annexin-v.556421?tab=product_details
Anti-mouse CD3-Ultra-LEAF Purified, 145-2C11, 100339, BioLegend, 5µg/ml
https://www.biolegend.com/en-gb/products/ultra-leaf-purified-anti-mouse-cd3epsilon-antibody-7722
Anti-mouse CD28-Ultra-LEAF Purified, 37.51, 102115, BioLegend, 2µg/ml
https://www.biolegend.com/en-gb/products/ultra-leaf-purified-anti-mouse-cd28-antibody-7733
Anti-mouse IL-2-Functional Grade, S4B6, 16-7020-85, Invitrogen, 10µg/ml
https://www.thermofisher.com/antibody/product/IL-2-Antibody-clone-S4B6-Monoclonal/16-7020-85
Anti-mouse PD-1-InVivoMAb, RMP1.14, BE0146, Bio X Cell, 10µg/ml
https://bioxcell.com/invivomab-anti-mouse-pd-1-cd279-be0146
Anti-human CD3-Ultra-LEAF Purified, OKT3, 317325, BioLegend, 0.5µg/ml
https://www.biolegend.com/en-us/products/ultra-leaf-purified-anti-human-cd3-antibody-7745
Anti-human PD-L1-Ultra-LEAF Purified, 29E.2A3, 329715, BioLegend, 20µg/ml
https://www.biolegend.com/en-us/products/ultra-leaf-purified-anti-human-cd274-b7-h1-pd-l1-antibody-7746
Mouse Isotype Control IgG2b-Ultra-LEAF Purified, MPC-11, 400347, Biolegend, 20µg/ml
https://www.biolegend.com/en-us/products/ultra-leaf-purified-mouse-igg2b-kappa-isotype-ctrl-8098
Anti-mouse CD153, RM153, AB01089-2-0-VXM, 2BScientific, 0.1mg/dose
https://www.2bscientific.com/products/absolute-antibody/ab01089-2-0-vxm/anti-cd153-rm153
Rat Isotype control IgG2a-InVivoMAb, 2A3, BE0089, Bio X Cell, 0.1mg/dose
https://bioxcell.com/invivomab-rat-igg2a-isotype-control-anti-trinitrophenol-be0089

Eukaryotic cell lines

Policy information about cell lines and Sex and Gender in Research

Cell line source(s): The B16F10 cell line was obtained from Dr. Ethan Shevach laboratory at NIAID, NIH, USA, with the original commercial source being ATCC (catalog number CRL-6475).

Authentication: The cell line was not authenticated.

Mycoplasma contamination: We can confirm the cell line was negative for mycoplasm.

Commonly misidentified lines (See ICLAC register): No commonly misidentifed lines were used in this study.

Animals and other research organisms

Policy information about studies involving animals; ARRIVE guidelines recommended for reporting animal research, and Sex and Gender in Research

Laboratory animals: All animals used in this study were C57BL/6 and the mouse strains were as follows: C57BL/6, CD45.1 C57BL/6, B6.Pd1-/-, B6.Foxp3RFP, B6.Pd1-/-Foxp3RFP, B6.Foxp3ERT2-Cre-eGFP, Pd1fl/fl-DTR-tdTomato, B6. Foxp3ERT2-Cre-eGFP;Pd1fl/fl-DTR-tdTomato and B6.Rag1-/-. All experimental animals were 8 to 12 weeks of age.

Wild animals: No wild animals were used in this study.

Reporting on sex: Both males and females were used as per MRC funding guidelines.

Field-collected samples	No field-collected samples were used in this study.
Ethics oversight	The animal work was performed under a Home Office Approved project license to Dr. Amaranth. PPL No: PBFB610F9. Protocol No: 1, 2 and 6.

Note that full information on the approval of the study protocol must also be provided in the manuscript.

Plants

Seed stocks	not applicable
Novel plant genotypes	not applicable
Authentication	not applicable

Flow Cytometry

Plots

Confirm that:

☒ The axis labels state the marker and fluorochrome used (e.g. CD4-FITC).

☒ The axis scales are clearly visible. Include numbers along axes only for bottom left plot of group (a 'group' is an analysis of identical markers).

☒ All plots are contour plots with outliers or pseudocolor plots.

☒ A numerical value for number of cells or percentage (with statistics) is provided.

Methodology

Sample preparation	All immune cells were processed using standardised procedures and expanded within Methods section.
Instrument	FACS Aria or FACS symphony was used to collect data
Software	FlowJo v10.7.1 was used for analysis. SPICE v6.1 software was used for boolean analysis
Cell population abundance	Routinely at least 10,000 FoxP3+ cells were captured for analysis.
Gating strategy	Gating strategy is outlined within each figure. Routinely, a fsc vs ssc gate was drawn, followed by singlet gating. Then a live cell gating was performed with fixable live/dead. Then CD4+ T cells were gated and then Foxp3 positive cell gating was used to measure coreceptors.

☒ Tick this box to confirm that a figure exemplifying the gating strategy is provided in the Supplementary Information.
